# Wnt, glucocorticoid and cellular prion protein cooperate to drive a mesenchymal phenotype with poor prognosis in colon cancer

**DOI:** 10.1186/s12967-024-05164-0

**Published:** 2024-04-08

**Authors:** Sophie Mouillet-Richard, Angélique Gougelet, Bruno Passet, Camille Brochard, Delphine Le Corre, Caterina Luana Pitasi, Camille Joubel, Marine Sroussi, Claire Gallois, Julien Lavergne, Johan Castille, Marthe Vilotte, Nathalie Daniel-Carlier, Camilla Pilati, Aurélien de Reyniès, Fatima Djouadi, Sabine Colnot, Thierry André, Julien Taieb, Jean-Luc Vilotte, Béatrice Romagnolo, Pierre Laurent-Puig

**Affiliations:** 1 Centre de Recherche Des Cordeliers, INSERM, Sorbonne Université, Université Paris Cité, 75006 Paris, France; 2 University of Paris-Saclay, INRAE, AgroParisTech, UMR1313 GABI, 78350 Jouy-en-Josas, France; 3 Institut du Cancer Paris CARPEM, APHP, Department of Pathology, APHP.Centre-Université Paris Cité, Hôpital Européen G. Pompidou, Paris, France; 4 Université Paris Cité, Institut Cochin, Inserm, CNRS, F-75014 Paris, France; 5 Equipe Labellisée Ligue Nationale Contre Le Cancer, Paris, France; 6 Institut du Cancer Paris CARPEM, APHP, Hepatogastroenterology and GI Oncology Department, APHP.Centre-Université Paris Cité, Hôpital Européen G. Pompidou, Paris, France; 7 Histology, Imaging and Cytometry Center (CHIC), Paris, France; 8 Saint-Antoine Hospital, INSERM, Unité Mixte de Recherche Scientifique 938, Sorbonne Université, Paris, France; 9 Institut du Cancer Paris CARPEM, APHP, Department of Biology, APHP.Centre-Université Paris Cité, Hôpital Européen G. Pompidou, Paris, France

**Keywords:** Colon cancer, Prion protein, Wnt-β-catenin, Glucocorticoid receptor, Molecular classification

## Abstract

**Background:**

The mesenchymal subtype of colorectal cancer (CRC), associated with poor prognosis, is characterized by abundant expression of the cellular prion protein PrP^C^, which represents a candidate therapeutic target. How PrP^C^ is induced in CRC remains elusive. This study aims to elucidate the signaling pathways governing PrP^C^ expression and to shed light on the gene regulatory networks linked to PrP^C^.

**Methods:**

We performed in silico analyses on diverse datasets of in vitro, ex vivo and in vivo models of mouse CRC and patient cohorts. We mined ChIPseq studies and performed promoter analysis. CRC cell lines were manipulated through genetic and pharmacological approaches. We created mice combining conditional inactivation of *Apc* in intestinal epithelial cells and overexpression of the human prion protein gene *PRNP*. Bio-informatic analyses were carried out in two randomized control trials totalizing over 3000 CRC patients.

**Results:**

In silico analyses combined with cell-based assays identified the Wnt-β-catenin and glucocorticoid pathways as upstream regulators of *PRNP* expression, with subtle differences between mouse and human. We uncover multiple feedback loops between PrP^C^ and these two pathways, which translate into an aggravation of CRC pathogenesis in mouse. In stage III CRC patients, the signature defined by *PRNP*-*CTNNB1*-*NR3C1*, encoding PrP^C^, β-catenin and the glucocorticoid receptor respectively, is overrepresented in the poor-prognosis, mesenchymal subtype and associates with reduced time to recurrence.

**Conclusions:**

An unleashed PrP^C^-dependent vicious circle is pathognomonic of poor prognosis, mesenchymal CRC. Patients from this aggressive subtype of CRC may benefit from therapies targeting the *PRNP*-*CTNNB1*-*NR3C1* axis.

**Supplementary Information:**

The online version contains supplementary material available at 10.1186/s12967-024-05164-0.

## Background

With over 900,000 deaths in 2020, colorectal cancer (CRC) remains the third most frequent cancer and the second cause of cancer-related deaths worldwide [1]. Despite tremendous progress in screening, diagnosis and therapy, the 5-years relative survival rate remains 65% (all disease stages), and even drops below 15% when patients are diagnosed at the metastatic stage (stage IV). Achieving a better understanding of the complex molecular mechanisms that orchestrate CRC pathogenesis still remains among the greatest challenges to improve therapeutic strategies.

Ever since the seminal identification of *APC* gene mutations associated with familial adenomatous polyposis over 30 years ago [2], the Wnt-β-catenin has remained the top-most scrutinized signaling pathway in CRC [3]. Around 90% of CRC cases have somatic mutations in the *APC* gene or other components of the Wnt pathway [3]. Although disturbed Wnt signaling is a constant feature in CRC, our knowledge of the co-regulatory pathways that cooperate with Wnt-β-catenin to promote CRC progression or that dictate specific transcriptional programs according to CRC molecular subtypes is far from complete. Indeed, the consensus molecular classification of CRC enables to divide tumors into one major or a combination of consensus molecular subtypes (CMS) based on bulk transcriptome profiling [4, 5]. Because *APC* mutations are prevalent in all CMS, although less frequent in the CMS1 subtype that corresponds to hypermutated and microsatellite unstable tumors [4], it can be surmised that CMS-specific signaling cascades orientate the Wnt response. In the CMS4 subtype that is associated with dismal prognosis, two pathways, namely TGFβ [6] and YAP/TAZ [7], have gathered special interest. These pathways are well known to crosstalk with Wnt-β-catenin signaling [8], and, therefore, are good candidates for influencing the Wnt-β-catenin output. Recently, we documented that the cellular prion protein PrP^C^, encoded by the *PRNP* gene, is an upstream regulator of these two major pathways in CMS4 CRC [9], and that its targeting is a promising approach to treat CMS4 patients [10]. Long confined to the field of neurodegenerative diseases, PrP^C^ is now attracting a great deal of interest in cancer research [11]. Its location at the cell membrane [12], coupled to its capacity to instruct downstream cell signaling events [13] make it an ideal candidate for fine-tuning the cellular response to environmental signals. One unanswered question relates to the induction of *PRNP* expression along CRC initiation and progression. By integrating in silico analyses on mouse and human datasets with cell-based experiments, we provide evidence that *PRNP* expression is jointly controlled by the Wnt and glucocorticoid signaling pathways. Using mice that combine *Apc* inactivation in intestinal epithelial cells and *PRNP* overexpression, we uncover a positive Wnt-PrP^C^ feedback loop that unleashes a vicious circle. Analyses carried out in several mouse models of β-catenin-driven liver cancer corroborated the occurrence of a Wnt-PrP^C^ axis. Our data further point to species differences in the regulation of *PRNP* expression, with glucocorticoid signaling playing a critical role in human as compared to mouse. Finally, we show in two randomized clinical trials (RCT) of stage III CRC, altogether encompassing over 3000 patients, that the PrP^C^-dependent axis is pathognomonic of the CMS4 subtype and is associated with dismal prognosis.

## Methods

### Gene expression analyses

The following datasets were retrieved from public sources: GSE200908 [14], GSE208372 [15], GSE167008 [16], GSE20916 [17], GSE8671 [18], GSE4183 [19], GSE39852 [20], GSE11406 [21], PRJEB44400 [22]. Due to missing values, some analyses could not be performed with the GSE20916 dataset. Kinetic RNAseq data from sorted Apc^∆hep^ hepatocytes are deposited on GEO with accession number GSE210482 [23].

### Patients cohorts and analyses

The IDEA-France cohort is composed of 1248 patients having signed informed consent within a phase III randomized trial comparing 3 months to 6 months of mFOLFOX6 (infusional fluorouracil, leucovorin, and oxaliplatin) or CAPOX (capecitabine, oxaliplatin) after curative resection of stage III CC [24], and for which we obtained RNAseq on punch biopsies, as described in [25].

The PETACC8 cohort is composed of 1733 patients having signed informed consent within a phase III randomized trial comparing FOLFOX4 (infusional fluorouracil, leucovorin, and oxaliplatin) to FOLFOX4 + cetuximab in 2550 patients after curative resection of stage III CC [26], and for which we obtained RNAseq on macrodissected formalin-fixed, paraffin-embedded (FFPE) tissue sections, as described in [25].

The demographics of the IDEA-France and the PETACC8 cohorts are summarized in Additional file 1: Tables S1 and S2, respectively.

3’RNAseq and bioinformatics analyses are detailed in Additional file 1.

For survival analyses, the optimal cutpoint value was determined to predict time to recurrence (TTR), using the surv_cutpoint function from R package survminer. TTR analyses were performed using the coxph function of the survival R package. All analyses were carried out using R studio (version 4.2.2).

### Statistical analysis

RNAseq datasets (GSE200908, GSE208372, GSE167008, PRJEB44400) were analyzed using the DESeq2 package version 1.38.3. All analyses were performed with R studio 4.2.2. All statistical analyses were performed in R studio (version 4.2.2) using the stat_compare_means function from the ggpubr package. Correlation analyses were performed in R studio (version 4.2.2) using the stat_cor function from the ggpubr package. The results from experimental data in cell lines are reported as the means ± standard errors of the means (s.e.m.) with graphs generated using GraphPad PRISM version 9.4.1. Analyses involving two groups were carried out using the Shapiro test followed by Student’s t test or Mann–Whitney rank-sum test according to normality. Results from RT-qPCR in mouse tissue and RNA analysis in public datasets or patient cohorts are expressed as median and interquartile range with graphs generated with ggplot2 in R studio. Statistical analysis was performed using the Mann–Whitney rank-sum test for two groups or one-way ANOVA followed by Wilcoxon rank-sum tests with Holm’s correction for multiple comparisons for > 2 groups.

Additional materials and methods are described in Additional file 1.

## Results

### PRNP is a target of Wnt-β-catenin signaling in various models of β-catenin-activated intestinal tumors

**Fig. 1:**
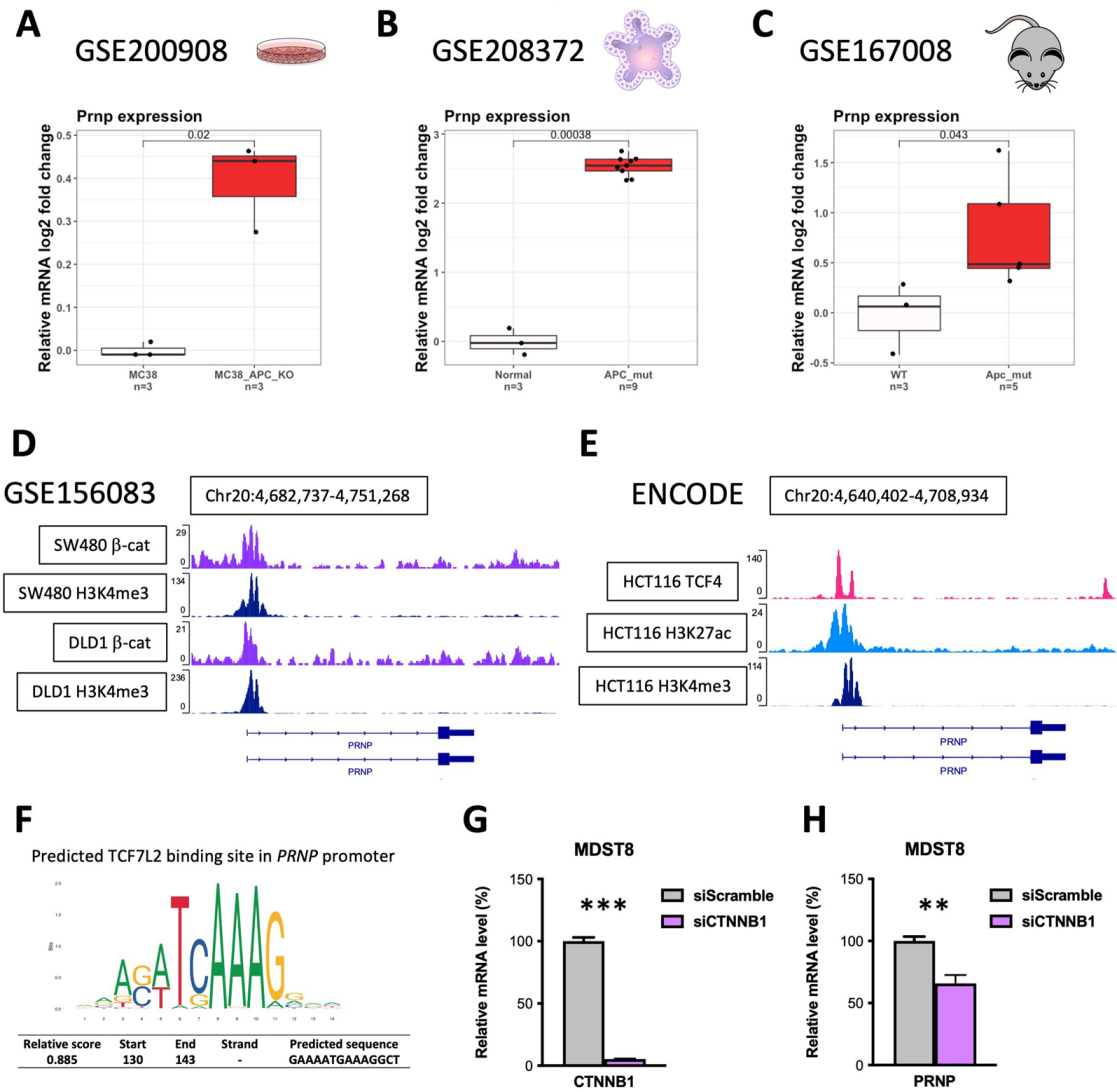
The *PRNP* gene is a target of Wnt-β-catenin signaling in colon cancer models. **A–C** Analysis of the GSE200908 (**A**) GSE208372 (**B**) and GSE167008 (**C**) datasets reveals increased *Prnp* expression in cellular, organoid and in vivo mouse models of Apc inactivation. **D** Analysis of the GSE156083 ChIPseq dataset reveals enrichment of β-catenin and the H3K4me3 active histone mark at the promoter of the *PRNP* gene in SW480 and DLD-1 human colon cancer cell lines. **E** Analysis from the ENCODE database reveals binding of the TCF7L2-encoded TCF4 factor, together with the H3K27ac and H3K4me3 active histone marks at the promoter of the *PRNP* gene in the HCT116 human colon cancer cell line. **F** Predicted TCF7L2 binding site within the human *PRNP* gene promoter. **G, H** Relative mRNA levels of *CTNNB1* (**G**) and *PRNP* (**H**) in *CTNNB1*-silenced versus control MDST8 cells, as determined in qPCR analysis. Results are expressed as means of *n* = 2 independent triplicates of cell preparations ± s.e.m. (***p < 0.001, **p < 0.01, Student’s t-test)

A still unresolved question regarding the overexpression of the cellular prion protein in colon cancer relates to the molecular pathways involved. By interrogating public datasets, we found that Apc inactivation in several mouse experimental paradigms, i.e. in the murine MC38 colon cancer cell line (GSE200908 [14]), mouse colonic tumor organoids (GSE208372 [15]), or mouse intestinal tumors (GSE167008 [16]), was systematically associated with *Prnp* gene upregulation (Fig. 1A–C). Assuming that *PRNP* might be a target of the canonical Wnt-β-catenin pathway, we subsequently mined ChIPseq datasets for β-catenin binding. As shown in Fig. 1D, we found that the *PRNP* promoter is occupied by β-catenin and harbors the active H3K4me3 mark (H3 histone tri-methylated on Lysine 4) in SW480 and DLD1 colon cancer cells in the GSE156083 dataset [27]. Likewise, searching the ENCODE database, we found that the *PRNP* promoter is bound by the *TCF7L2* gene-encoded TCF4 transcription factor, the main β-catenin co-factor in colon and liver, as well as H3K27ac (H3 histone acetylated on Lysine 27) and H3K4me3, which both mark active chromatin, in HCT116 colon cancer cells (Fig. 1E). We further identified one TCF4 binding site in the promoter of *PRNP* by analyzing predicted transcription factor binding sites using the JASPAR database [28] (Fig. 1F). In agreement with the above data, *PRNP* expression was significantly reduced in β-catenin-silenced MDST8 colon cancer cells (Fig. 1G, H). Altogether these data show that β-catenin/TCF4 bind Wnt-responsive element (WRE)-encompassing enhancers specifically active in *PRNP* in human β-catenin activated tumor cells.

### PRNP is a target of the glucorticoid receptor in CRC cells

**Fig. 2:**
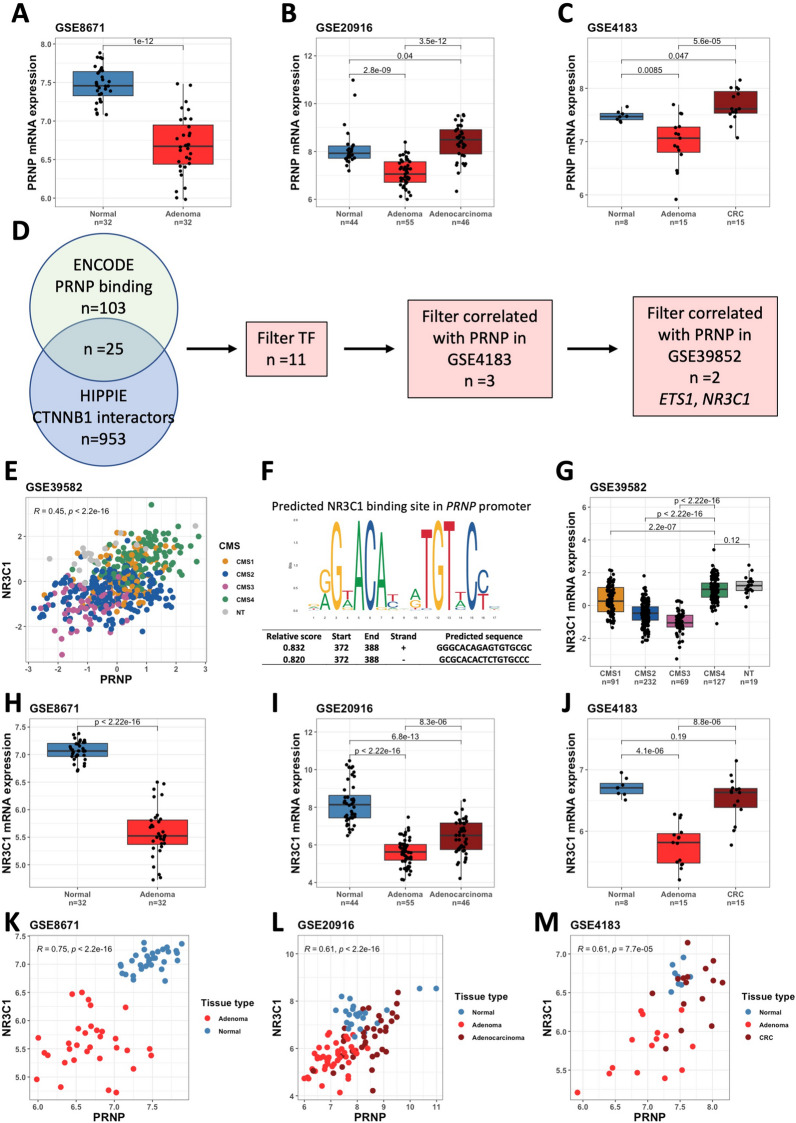
The GR encoded by the *NR3C1* gene is predicted to regulate *PRNP* gene expression in human CRC. **A–C** Analysis of the GSE8671 (**A**) GSE20916 (**B**) and GSE4183 (**C**) datasets reveals decreased *PRNP* expression across the normal to adenoma sequence, followed by an increase from the adenoma to carcinoma sequence in human CRC. **D** Schematic representation of the selection of *ETS1* and *NR3C1* as candidate transcription factors regulating the expression of *PRNP* in human CRC. **E** Scatter plot showing the correlation between *PRNP* and *NR3C1* mRNA levels in the GSE39582 dataset of human CRC. **F** Predicted NR3C1 binding sites within the human *PRNP* gene promoter. **G** Relative *NR3C1* mRNA levels according to the CMS classification in the GSE39582 dataset of human CRC. NT = non tumor. **H–J** Analysis of the GSE8671 (**H**) GSE20916 (**I**) and GSE4183 (**J**) datasets reveals decreased *NR3C1* expression across the normal to adenoma sequence, followed by an increase from the adenoma to carcinoma sequence in human CRC. **K–M** Scatter plots showing the correlation between *PRNP* and *NR3C1* mRNA levels in GSE8671 (**K**) GSE20916 (**L**) and GSE4183 (**M**) datasets

Turning to CRC patients, we analyzed the expression profile of *PRNP* along disease progression from normal to adenoma to carcinoma. In three distinct datasets GSE8671 [18], GSE20916 [17] and GSE4183 [19], we recurrently found decreased expression of *PRNP* transcripts in adenoma versus normal tissue (Fig. 2A–C), contrasting with the increase in the expression of several Wnt target genes including *AXIN2* [18]. On another hand, colon cancer tissue exhibited higher amounts of *PRNP* as compared to both adenoma and normal tissue (Fig. 2B, C), confirming the previous report by de Wit et al. that PrP^C^ is induced along the adenoma-carcinoma sequence [29]. It is well established that the recruitment of β-catenin to target genes is not always sufficient to induce transcription and that β-catenin-dependent transcriptional regulation is highly context-specific and often involves co-factors [30]. Because the Wnt pathway is switched on in adenomas but is not accompanied by an increase in *PRNP* mRNAs, in contrast to our observations in mouse models, we thus surmised that β-catenin may cooperate with (an)other regulatory factor(s) to promote *PRNP* transcription in human cells. To identify potential candidates, we selected the transcription factors (TF) that fulfil the following criteria: (1) binding to the *PRNP* gene in the ENCODE database and (2) featuring in the HIPPIE (Human Integrated Protein–Protein Interaction rEference, [31]) database of β-catenin interactors. We further filtered the list of 11 candidates to keep TF whose expression is significantly correlated to that of *PRNP* in the two patient datasets GSE4183 and GSE39852 [20] (Fig. 2D). *NR3C1*, which encodes the glucocorticoid receptor, GR, was more strongly correlated with *PRNP* in the GSE39852 dataset than was *ETS1* (*R* = 0.45 versus *R* = 0.32) and was selected for further analysis (Fig. 2E). Using the JASPAR database, we identified a consensus palindromic binding motif for NR3C1 in the *PRNP* promoter (Fig. 2F). Of note, this sequence is not conserved in the mouse *Prnp* promoter sequence (Additional file 1: Fig. S1A), although another GR responsive element (GRE) is found further upstream, according to JASPAR analysis (Additional file 1: Fig. S1B), in contrast to the WRE that is conserved from human to mouse (Additional file 1: Fig. S1A). The expression profile of *NR3C1* is highly compatible with a GR-dependent regulation of *PRNP* gene expression since it is most abundantly expressed in the CMS4 subtype of CRC (Fig. 2G), it globally follows the pattern of *PRNP* expression along the normal to adenoma to adenocarcinoma sequence in patients (Fig. 2H–J), and *NR3C1* and *PRNP* levels are significantly correlated in various datasets (Fig. 2K–M).

**Fig. 3:**
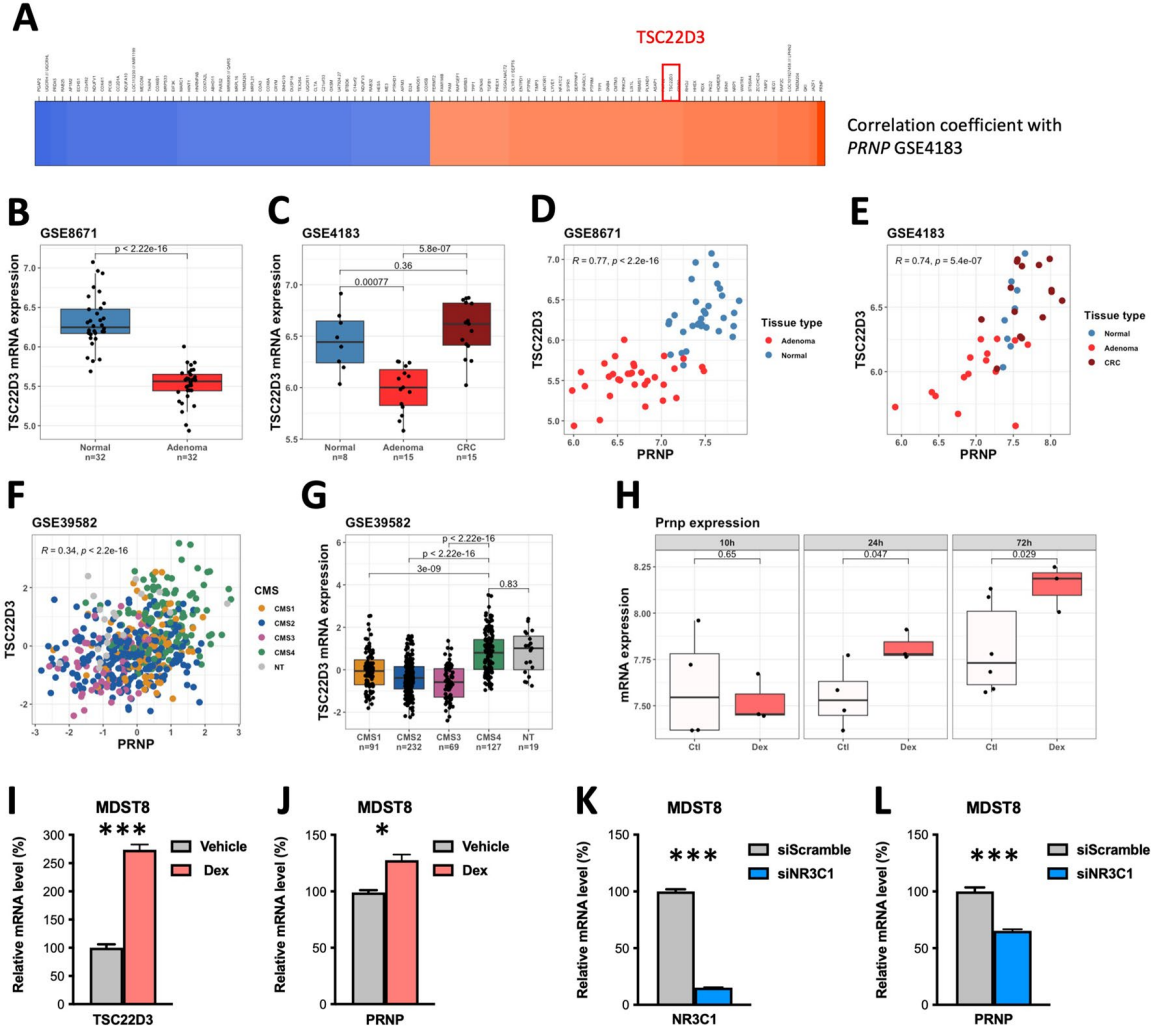
*PRNP* gene expression is regulated by the GR in human CRC. **A** Heatmap showing the top 20 positively (in red) and negatively (in blue) correlated genes to *PRNP* expression in the GSE4183 dataset. Highlighted is the GR target *TSC22D3*. **B, C** Analysis of the GSE8671 (**B**) and GSE4183 (**C**) datasets reveals decreased *TSC22D3* expression across the normal to adenoma sequence, followed by an increase from the adenoma to carcinoma sequence in human CRC. **D, E** Scatter plots showing the correlation between *PRNP* and *TSC22D3* mRNA levels in GSE8671 (**D**) and GSE4183 (**E**) datasets. **F** Scatter plot showing the correlation between *PRNP* and *TSC22D3* mRNA levels in the GSE39582 dataset of human CRC. **G** Relative *TSC22D3* mRNA levels according to the CMS classification in the GSE39582 dataset of human CRC. NT = non tumor. **H** Boxplots showing the time-dependent increase in *Prnp* expression in oligodendrocyte progenitor cells (OPCs) exposed to Dexamethasone (Dex) (GSE11406 dataset). **I, J** Relative mRNA levels of *TSC22D3* (**I**) and *PRNP* (**J**) in MDST8 cells exposed to Dex (1 µM, 24 h) versus vehicle (water), as determined in qPCR analysis. *n* = 3 cell preparations per condition. (***p < 0.001, *p < 0.05, Student’s t-test). **K, L** Relative mRNA levels of *NR3C1* (**K**) and *PRNP* (**L**) in *NR3C1*-silenced versus control MDST8 cells, as determined in qPCR analysis. *n* = 2 independent triplicates of cell preparations. (***p < 0.001, Student’s t-test)

Because the expression of *NR3C1* mRNA cannot be considered as a readout of GR activation, we reasoned that if *PRNP* is indeed a GR target, then it should be co-expressed with well-established GR-regulated genes. Indeed, *TSC22D3*, also known as Glucocorticoid-Inducible Leucine Zipper (*GILZ*), features among the top-50 *PRNP* positively correlated genes in the GSE4183 dataset (Fig. 3A). As observed with *NR3C1*, *TSC22D3* transcripts are reduced in adenoma versus normal tissue but further induced at the adenoma-to-carcinoma progression (Fig. 3B, C); they strongly correlate with *PRNP* levels, including in the large GSE39582 dataset (Fig. 3D–F) and are enriched in CMS4 CRC (Fig. 3G). We then assessed whether *PRNP* transcription is induced upon GR activation. In the GSE11406 dataset [21], *Prnp* levels were induced in oligodendrocyte progenitor cells (OPC) after exposure to Dexamethasone (Dex) in a time-dependent manner (Fig. 3H). Likewise, we found increased expression of *PRNP* in the MDST8 CRC cell line upon treatment with Dex (1 µM, 24 h), accompanying increased *TSC22D3* expression (Fig. 3I-J). Reciprocally, *PRNP* levels were reduced in *NR3C1*-silenced MDST8 cells (Fig. 3K, L). Combined with our previous report that PrP^C^ controls the expression of NR3C1 in CRC cells [9], these data provide evidence for a positive feedback loop linking PrP^C^ to the GR and suggest that the GR may cooperate with β-catenin to regulate *PRNP* gene expression in CRC.

### ***PrP***^***C***^*** overexpression boosts Wnt signaling in mouse intestine***

According to our previous findings and the above data, PrP^C^ sustains positive feedback loops with multiple effectors in colon cancer cells: TGFβ1 [9], but also the integrin linked kinase (ILK) [32], the amyloid precursor protein (APP)-derived Aβ [10], or the GR (cf above). This prompted us to investigate whether this would also apply to the Wnt pathway. This hypothesis is notably supported by the demonstration by Besnier et al. that PrP^C^ interacts with β-catenin and its cofactor TCF7L2 and upregulates the transcriptional activity of the β-catenin/TCF7L2 complex in SW480 cells [33]. Corroborating this finding, we found that PrP^C^ silencing in MDST8 or SW480 colon cancer cells reduced the levels of *AXIN2* mRNA, while its overexpression in LoVo cells was associated to an opposite increase in *AXIN2* transcripts (Additional file 1: Fig. S2). To test our hypothesis, we sought to generate mice combining inducible Apc inactivation with PrP^C^ overexpression with the idea that a PrP^C^-Wnt positive feedback loop would translate into an increase in Wnt targets in normal tissue of PrP^C^ overexpressing mice and sustain a vicious circle amplifying the expression of PrP^C^ itself as well as its targets in Apc-mutant tumors. Mice containing a floxed mutant allele of the Apc gene (*Apc*^fl/+^) [34] as well as tamoxifen-dependent Cre-recombinase under the control of the Villin promoter (*Vill-cre*) [35], i.e. *VilCreER*^T2^*Apc*^fl/+^ mice [36], were crossed with mice overexpressing the human *PRNP* gene under its own regulatory sequences (through the use of a transgene composed of a large human genomic insert purified from a P1-derived artificial chromosome), in an endogenous mouse *Prnp*-null context (*PRNP*^+/+^*Prnp*^−/−^, hereafter referred to as *PRNP*^+/+^) [37], as outlined in Additional file 1: Fig. S3A. Importantly, the latter so-called tg650 mice have been extensively used in prion studies and were not reported to spontaneously overdevelop tumors compared to wild-type mice (personal communication JLV), indicating that PrP^C^ overexpression per se does not initiate tumorigenesis. On another hand, these mice moderately overexpress PrP^C^ in the heterozygous state (*PRNP*^+/−^) and overexpress PrP^C^ sixfold versus WT in the homozygous state (*PRNP*^+/+^) [37]. They further allow to capture regulatory events impacting *PRNP* expression, contrary to the tga20 mouse model that overexpresses murine *Prnp* (eightfold) in an unregulated fashion [38]. To induce Apc loss of function, *VilCreER*^T2^*Apc*^fl/+^-*PRNP*^+/−^ and *VilCreER*^T2^*Apc*^fl/+^-*PRNP*^+/+^ mice were treated with tamoxifen (see Materials and Methods) and maintained until they showed signs of intestinal illness. Several tamoxifen-untreated *VilCreER*^T2^*Apc*^fl/+^-*PRNP*^+/−^ or Cre-negative *Apc*^fl/+^-*PRNP*^+/−^ mice remained healthy at comparable time points, confirming that the loss of function of Apc is mandatory to initiate tumorigenesis in this mouse model (data not shown). We then harvested tissue from *VilCreER*^T2^*Apc*^fl/+^-*PRNP*^+/−^ and *VilCreER*^T2^*Apc*^fl/+^-*PRNP*^+/+^ mice and assessed the expression of several markers of interest through RT-qPCR. As shown in Additional file 1: Fig. S3B, *PRNP* levels were comparable in control (tamoxifen-untreated *VilCreER*^T2^*Apc*^fl/+^-*PRNP*^+/−^ or Cre-negative *Apc*^fl/+^-*PRNP*^+/−^) and tamoxifen-treated *VilCreER*^T2^*Apc*^fl/+^-*PRNP*^+/−^ normal tissue, indicating that *PRNP* gene expression is insensitive to tamoxifen treatment. Then, we compared the normal tissues of *PRNP*^+/−^ and *PRNP*^+/+^ mice. Elevated expression of *PRNP* in homozygous mice (Additional file 1: Fig. S4A) was accompanied by increased expression of a set of genes that we previously identified as PrP^C^ gene targets in colon cancer cells [10], namely *App*, *Bace1*, *Dkk3*, *Pdgfc* and *Tgfb1* (Additional file 1: Fig. S4B–F), thus validating our model as a robust *PRNP*-overexpressing paradigm. We further recorded increased expression of the canonical Wnt target genes *Axin2*, *Lgr5* and *Ccnd1* in homozygous *PRNP*^+/+^ mice versus their heterozygous counterparts (Additional file 1: Fig. S4G–I). As observed for *PRNP*, PrP^C^-dependent target genes (Additional file 1: Fig. S3C–G) and canonical Wnt target genes (Additional file 1: Fig. S3H–J) were not sensitive to tamoxifen treatment per se. As a whole, these data support our hypothesis that PrP^C^ boosts Wnt signaling.

### ***PrP***^***C***^*** overexpression aggravates oncogenic Apc-induced colon tumorigenesis***

**Fig. 4:**
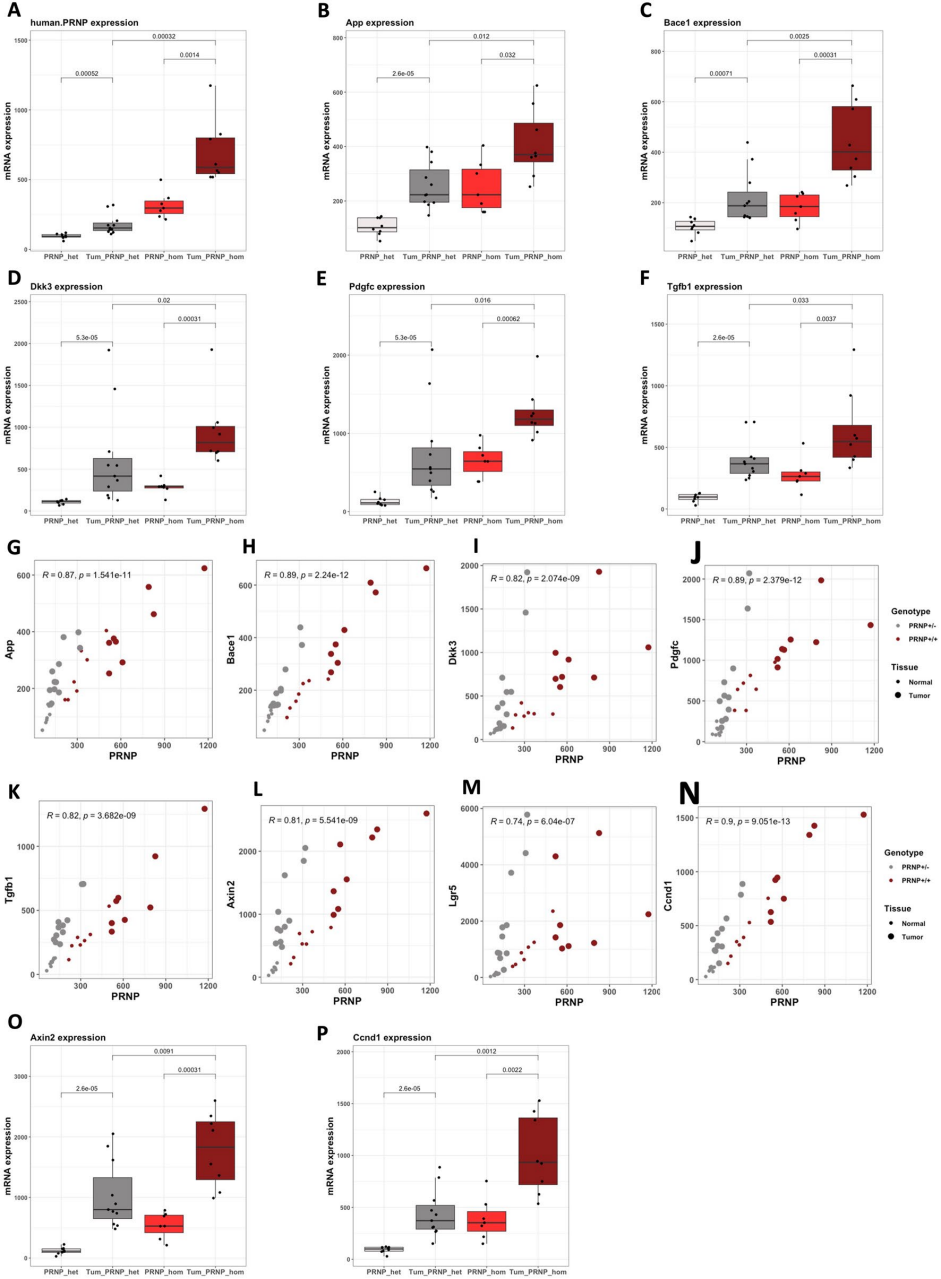
PrP^C^ sustains a vicious circle in an oncogenic Apc mouse model. **A–F** Boxplots showing the mRNA levels of human *PRNP* (**A**), and mouse *App* (**B**), *Bace1* (**C**), *Dkk3* (**D**), *Pdgfc* (**E**) and *Tgfb1* (**F**) in normal or tumor tissue from *VilCreER*^T2^*Apc*^fl/+^-*PRNP*^+/−^ (PRNP_het) or *VilCreER*^T2^*Apc*^fl/+^-*PRNP*^+/+^ (PRNP_hom) mice as measured through qRT-PCR. **G-N** Scatter plots showing the correlation between the mRNA levels of human *PRNP* and those of mouse *App* (**G**), *Bace1* (**H**), *Dkk3* (**I**), *Pdgfc* (**J**), *Tgfb1* (**K**), *Axin2* (**L**), *Lgr5* (**M**) and *Ccnd1* (**N**) in normal or tumor tissue from *VilCreER*^T2^*Apc*^fl/+^-*PRNP*^+/−^ or *VilCreER*^T2^*Apc*^fl/+^-*PRNP*^+/+^ mice. **O, P** Boxplots showing the mRNA levels of mouse *Axin2* (**O**) and *Ccnd1* (**P**) in normal or tumor tissue from *VilCreER*^T2^*Apc*^fl/+^-*PRNP*^+/−^ (PRNP_het) or *VilCreER*^T2^*Apc*^fl/+^-*PRNP*^+/+^ (PRNP_hom) mice as measured through qRT-PCR. Mice experiments were conducted as described in Additional file 1

**Fig. 5:**
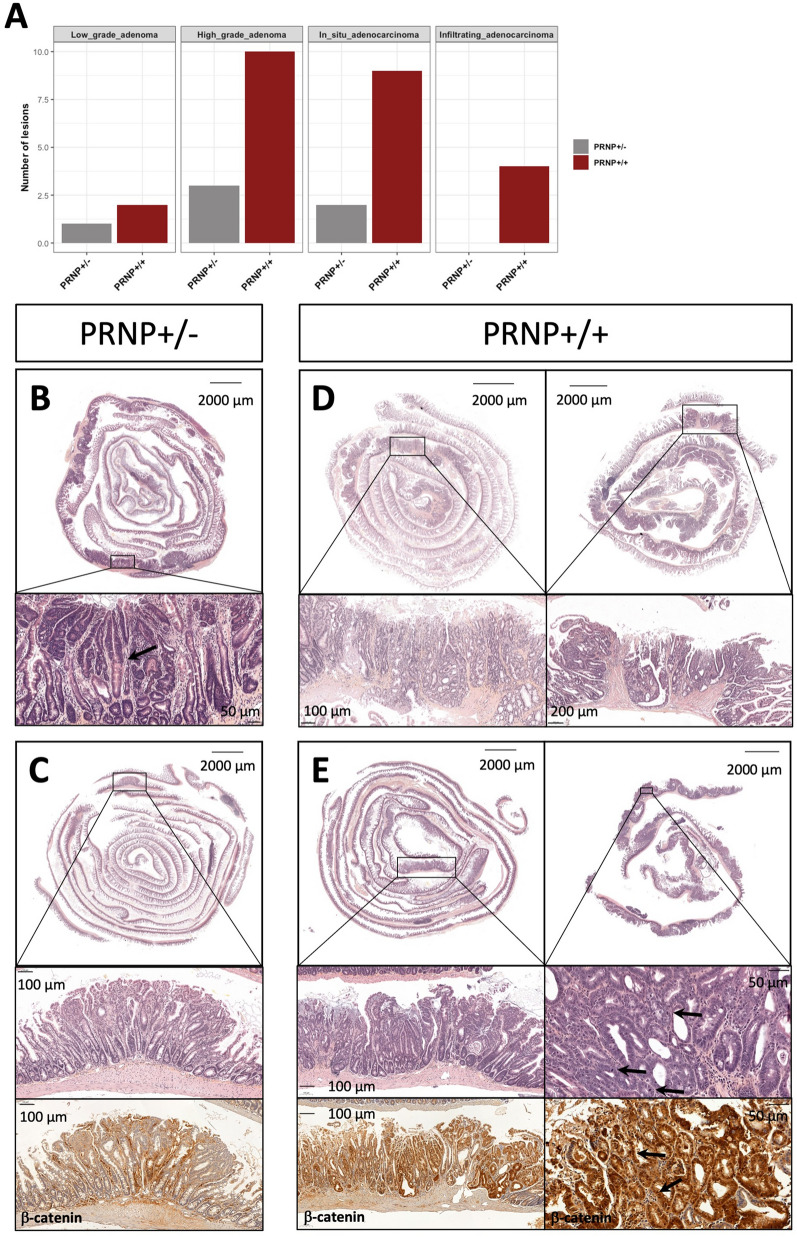
PrP^C^ overexpression aggravates the development of mutant Apc driven CRC in mouse. **A** Relative quantification of lesion types according to genotype. **B, C** H&E staining sections from *PRNP*^+/−^ mice, showing representative zones of high grade dysplasia (**B**, bottom panel) and low grade dysplasia (**c**, middle panel), as well as β-catenin staining (**C**, bottom panel). **D, E** H&E staining sections from *PRNP*^+/+^ mice, showing representative zones of infiltrating adenocarcinoma (**D**, bottom panels) and high grade dysplasia (**E**, middle panels), as well as β-catenin staining (**E**, bottom panels). Arrows indicate: a transition zone between normal cells and high grade dysplasia cells (**B**, bottom panel), mitoses (**E**, middle right panel), nuclear β-catenin (**E**, bottom right panel). Mice experiments were conducted as described in Additional file 1

We went on to examine the impact of PrP^C^ overexpression on the same set of genes in intestinal tumors. We first found that *PRNP* mRNA levels were elevated in intestinal tumors versus normal tissue, whatever the genetic status (hetero- or homozygous) of the mice (Fig. 4A). The mean *PRNP* levels measured in the tumors of *PRNP*^+/−^ mice however remained inferior to that measured in the normal tissue of *PRNP*^+/+^ mice. In the end, the mean *PRNP* mRNA level found in *PRNP*^+/+^ tumors reached 3.8 fold that of *PRNP*^+/−^ tumors. In agreement with the above findings, we also found that the expression of the PrP^C^ target genes *App*, *Bace1*, *Dkk3*, *Pdgfc* and *Tgfb1* were all increased in the tumor versus normal tissue of both types of mice, and that the tumor levels were higher in homozygous versus heterozygous mice (Fig. 4B–F). We actually found that the expression levels of these genes were highly correlated to those of *PRNP* (Fig. 4G–K). In our mouse model, we further found that the levels of the Wnt target genes *Axin2*, *Lgr5* and *Ccnd1* were also strongly correlated to those of *PRNP* (Fig. 4L–N). As a result, the mean levels of *Axin2* and *Ccdn1* were much higher in tumors from homozygous than heterozygous mice (1.7 fold and 2.3 fold for *Axin2* and *Ccdn1*, respectively, Fig. 4O, P). At histological examination, *VilCreER*^T2^*Apc*^fl/+^-*PRNP*^+/−^ mice exhibited a small number of adenomas or in situ adenocarcinomas (Fig. 5A–C), while *VilCreER*^T2^*Apc*^fl/+^-*PRNP*^+/+^ mice were found to harbor numerous high-grade adenomas, in situ and even infiltrating adenocarcinomas, (Fig. 5A and D–E) suggesting that the overexpression of PrP^C^ promotes colon cancer aggressiveness. In agreement, *PRNP*^+/+^ tumors exhibited intense nuclear or cytoplasmic β-catenin staining (Fig. 5E, lower panels) in contrast to *PRNP*^+/−^ tumors that present a lower accumulation of β-catenin (Fig. 5C, lower panel).

### PRNP expression is induced in mouse models of liver cancer with β-catenin activation

**Fig. 6:**
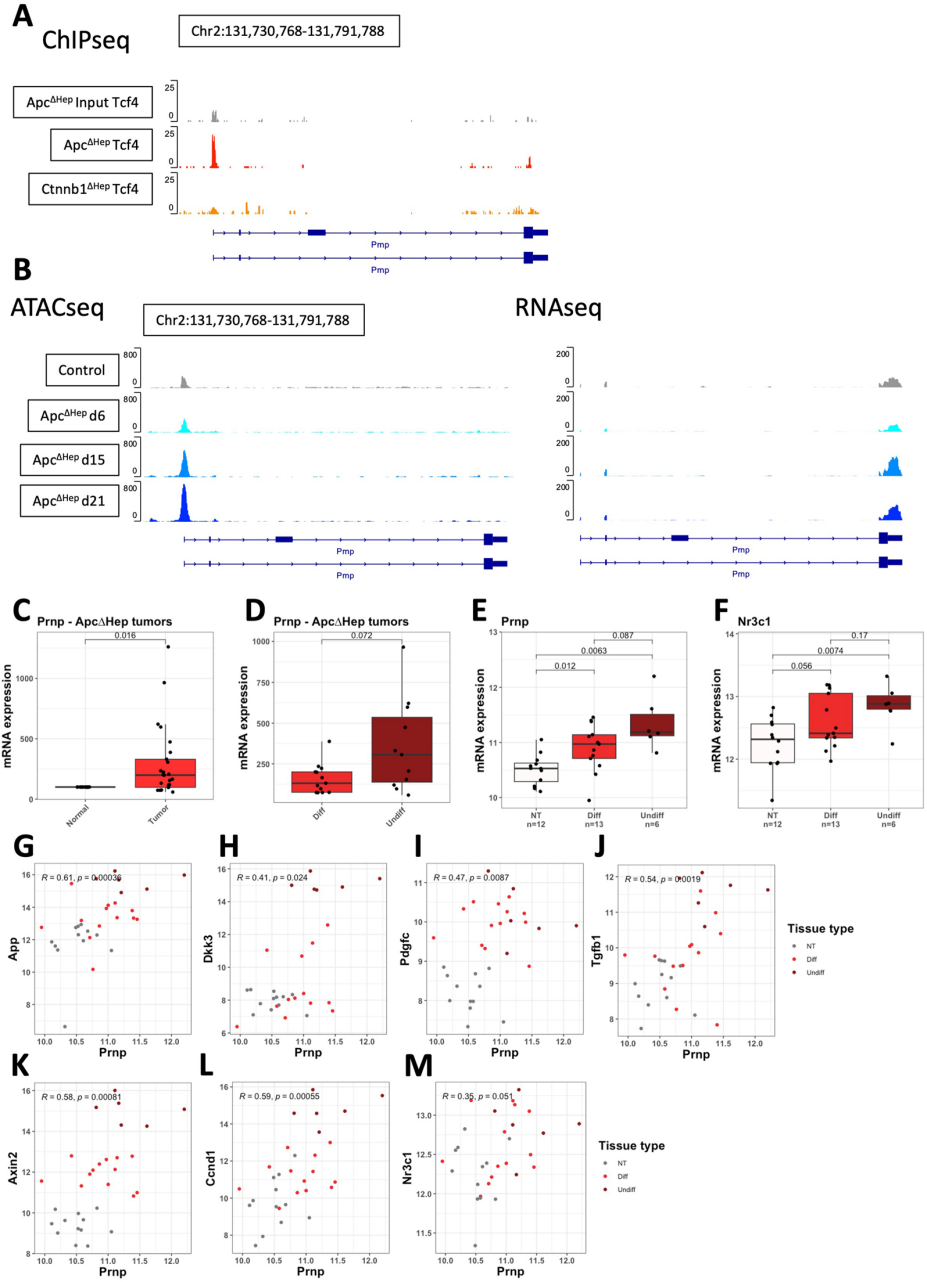
The *Prnp* gene is a target of Wnt-β-catenin signaling in mouse models of liver cancer. **A** Analysis of our GSE35213 ChIPseq dataset reveals enrichment of the Tcf7l2-encoded TCF4 transcription factor at the promoter of the *Prnp* gene in *Apc*-inactivated (Apc^∆hep^), but not control or *Ctnnb1*-deficient hepatocytes. **B** Analysis of our GSE242267 ATACseq dataset and our GSE210482 RNAseq showing the promoter accessibility (left) and RNA transcription (right) of the Prnp gene along the kinetics (days 6, 15 and 21) of *Apc* inactivation in hepatocytes as compared to GFP^−^ control. **C** Boxplots showing the mRNA levels of *Prnp* in Apc^∆hep^ tumor versus Apc^∆hep^ normal tissue, as measured through qRT-PCR. **D** Boxplots showing the mRNA levels of *Prnp* in undifferentiated versus differentiated liver tumors from Apc^∆hep^ mice, as measured through qRT-PCR. **E, F** Analysis of our PRJEB44400 dataset reveals increased *Prnp* (**E**) and *Nr3c1* (**F**) expression in differentiated and mostly undifferentiated liver tumors from mutant *Ctnnb1* and *Apc* mice. **G–M** Scatter plots showing the correlation between *Prnp* and *App* (**G**)*, Dkk3* (**H**)*, Pdgfc* (**I**)*, Tgfb1* (**J**)*, Axin2* (**K**)*, Ccnd1* (**L**) and *Nr3c1* (**M**) mRNA levels in the PRJEB44400 dataset

To possibly extend our observations, we turned to liver cancer models, in which activation of β-catenin is one the most frequent mutational event [39]. We first exploited a mouse model based on Cre-Lox inactivation of Apc in hepatocytes (Apc^∆hep^) that recapitulates both hepatocellular carcinoma and hepatoblastoma with aberrant β-catenin activation [22, 40, 41]. In the pre-tumoral setting, analysis of our ChIPseq data [41] indicated that *Tcf7l2*-encoded TCF4 bound to the *Prnp* promoter in hepatocytes shortly after β-catenin activation, which was not observed when β-catenin was invalidated (Fig. 6A), thus confirming our earlier data that TCF4 regulates *Prnp* expression through direct binding to its promoter region. In line with this, ATACseq analysis revealed a time-dependent opening of chromatin at the *Prnp* promoter in GFP + Apc^∆hep^-sorted hepatocytes, accompanied by *Prnp* transcription, as inferred through RNAseq (Fig. 6B). In qPCR experiments, we confirmed the induction of *Prnp* gene expression in tumors from Apc^∆hep^ mice (Fig. 6C), which was not observed in β-catenin-independent liver tumors induced by diethylnitrosamine (DEN) (Additional file 1: Fig. S5A). Of note, there was a tendency towards higher levels of *Prnp* in undifferentiated tumors, which resemble hepatoblastoma and display an epithelial to mesenchymal transition signature [22], as opposed to differentiated tumors that are similar to human hepatocellular carcinoma from G5-G6 groups (Fig. 6D). Similar results were obtained using RNAseq data from our previous study [22], combining liver tumors from either mutant *Ctnnb1* deleted of exon 3 obtained by in vivo CRISPR/Cas9 editing or Apc^∆hep^ mice (Fig. 6E). Interestingly, *Nr3c1* transcripts followed the same pattern of expression as *Prnp* (Fig. 6F), further reinforcing the link between PrP^C^ and glucocorticoid signaling found in the context of colon cancer (see above), and the two Wnt target genes *Axin2* and *Ccnd1* were higher in undifferentiated as compared to differentiated tumors (Additional file 1: Fig. S5C, D). Finally, in the same dataset, we found that *Prnp* mRNA levels were significantly correlated to those of *App*, *Dkk3*, *Pdgfc*, *Tgfb1*, *Axin2*, *Ccnd1* and *Nr3c1* (Fig. 6G–M). Altogether, these results provide further evidence that *Prnp* is a target of the Wnt-β-catenin pathway in multiple tissues and that it defines a signaling axis that is conserved from colon to liver cancer in mice.

### The PRNP-CTNNB1-NR3C1 axis defines a group of CRC patients with dismal prognosis

**Fig. 7:**
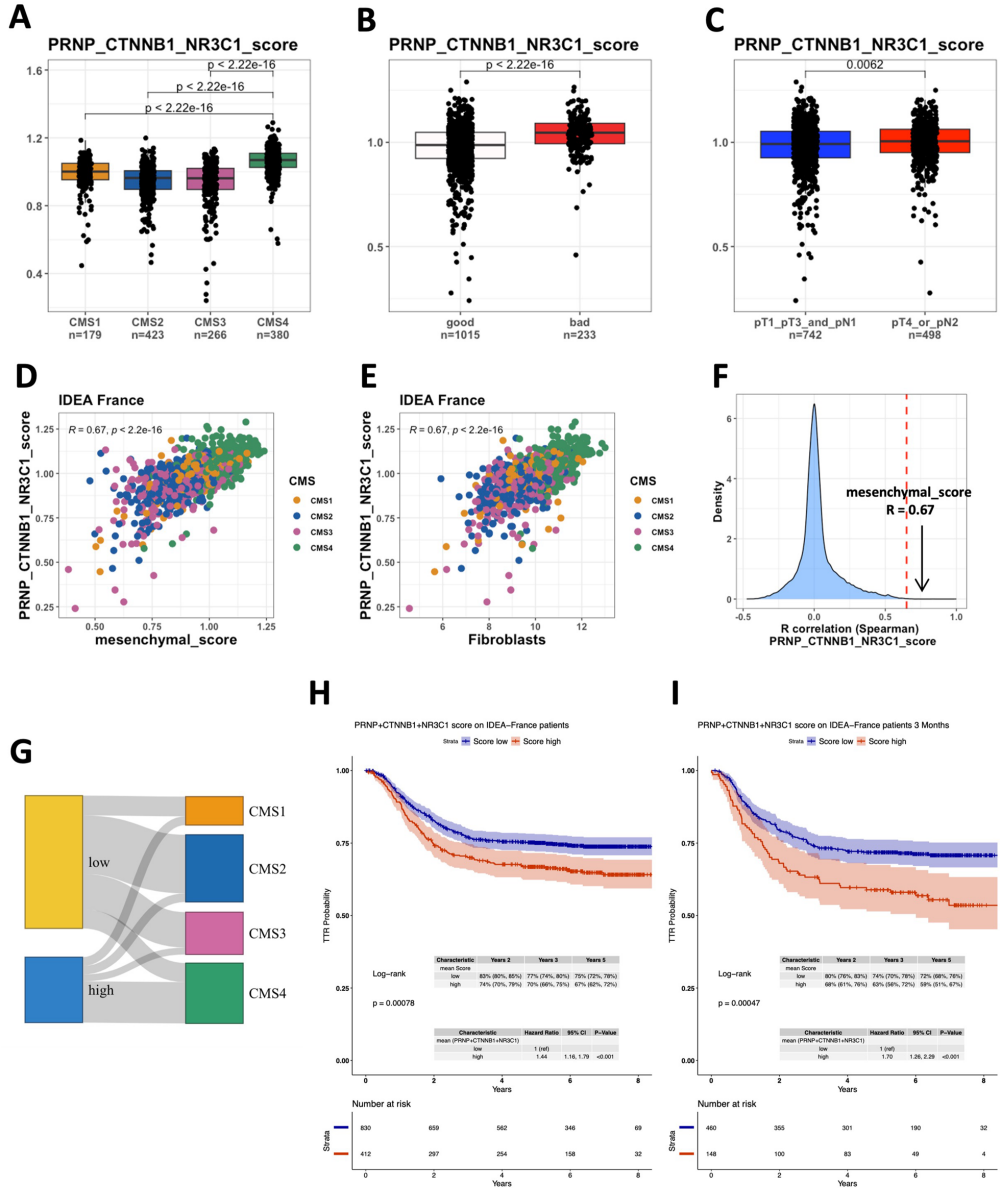
The *PRNP*-dependent axis is over-represented in poor-prognosis subtypes of CRC in the IDEA-France randomized clinical trial and predicts dismal outcome. **A–C** Relative *PRNP*-*CTNNB1*-*NR3C1* score according to the CMS classification (**A**), CMS combination (**B**) or TNM risk (**C**) in the IDEA France cohort. Patients with stage pT1-T3 and pN1 were classified as low TNM risk and those with pT4 and / or pN2 as high TNM risk. **D–F** Scatter plots showing the correlation between the *PRNP*-*CTNNB1*-*NR3C1* score and the mesenchymal (**D**) or the fibroblast (**E**) scores in the IDEA France cohort. **F** Density plot showing the distribution of correlation coefficients with the *PRNP*-*CTNNB1*-*NR3C1* score in the IDEA France cohort highlighting the top-ranked correlation with the mesenchymal score. **G** Sankey plots showing the correspondence between low or high *PRNP*-*CTNNB1*-*NR3C1* score and CMS subclasses. **H, I** Kaplan–Meier curves comparing time to recurrence in patients with low or high *PRNP*-*CTNNB1*-*NR3C1* score in the entire IDEA France cohort (**H**) or in the subset of patients having received chemotherapy during 3 months (**I**)

From the above data, we may hypothesize that, in CRC patients, β-catenin and glucocorticoid pathway activation cooperatively induce *PRNP* expression and may, as a consequence, instigate a detrimental vicious circle. Accordingly and in line with our previous findings [9, 10] and those illustrated in Fig. 2G and 3G, *PRNP*, *BACE1*, *DKK3*, *PDGFC*, *NR3C1* and *TSC22D3* levels were all enriched in the CMS4 subgroups of tumors from the IDEA France cohort (Fig. S6A-F), uniquely composed of stage III CRC cases [24]. However, this was not the case for the canonical Wnt targets *AXIN2* and *LGR5* (Additional file 1: Fig. S6G, H), in line with data reported in cell lines [42]. In addition, the expression levels of *BACE1*, *DKK3*, *PDGFC*, *NR3C1* and *TSC22D3* were all significantly correlated with those of *PRNP* (Additional file 1: Fig. S6I–M). Finally, we found that genes from this network were all enriched in the CMS combinations that we identified as associated with a notable dismal prognosis in our recent work [5] (Additional file 1: Fig. S6N–S). Again, opposite results were obtained for the two canonical Wnt targets *AXIN2* and *LGR5* (Additional file 1: Fig. S6T–U). Similar analyses in the PETACC8 cohort [26] yielded comparable results (Additional file 1: Fig. S7), although the correlations between *PRNP* and *BACE1* or *PDGFC* were modest as compared to those between *PRNP* and *DKK3*, *NR3C1* and *TSC22D3*, whose expression is majorly controlled by the GR (data not shown). The differences between the two cohorts may be accounted for by the different material used for 3’RNAseq (punch biopsies for the IDEA cohort and macro-dissected tissue sections for the PETACC8 cohort). Next, we calculated a *PRNP*-*CTNNB1*-*NR3C1* score and we found this score to be significantly higher in CMS4 patients from the IDEA France cohort (Fig. 7A). This score was also increased in the group of patients with a pejorative CMS combination (Fig. 7B) or in patients with high-risk disease (pT4 and/or N2) (Fig. 7C). Furthermore, major correlations were observed between the *PRNP*-*CTNNB1*-*NR3C1* score and a mesenchymal score derived from the study by de Reyniès et al. [43] (Fig. 7D), or a fibroblast score based on the MCP counter deconvolution algorithm [44] (Fig. 7E). As shown in Fig. 7F, the correlation between the mesenchymal and the *PRNP*-*CTNNB1*-*NR3C1* score featured among the highest in the IDEA-France cohort, arguing that the PrP^C^-Wnt-GR axis is a genuine hallmark of mesenchymal CRC.

A higher *PRNP*-*CTNNB1*-*NR3C1* score was also found in PETACC8 patients belonging to the CMS4 or the dismal prognosis CMS combination subgroups (Additional file 1: Fig. S8A, B). When we dichotomized patients from the IDEA France cohort according to this *PRNP*-*CTNNB1*-*NR3C1* score, we observed that patients with a high score segregated into the CMS4 subgroup (Fig. 7G and Additional file 1: Table S3) and were significantly associated with the poor CMS combination or high TNM risk subgroups (Additional file 1: Table S3). Patients with a high *PRNP*-*CTNNB1*-*NR3C1* score in the PETACC8 cohort were also more represented in the CMS4 and pejorative CMS combination groups (Additional file 1: Fig. S8D). Finally, a high *PRNP*-*CTNNB1*-*NR3C1* score in the IDEA France cohort was associated with a significantly shorter time to recurrence than a low score (HR = 1.44, CI = 1.16–1.79, *p* < 0.001) (Fig. 7H), which was even more apparent within the group of patients who received chemotherapy for 3 months instead of 6 months (HR = 1.70, CI = 1.26–2.29, *p* < 0.001) (Fig. 7I). This was also true when considering only patients having been treated with FOLFOX (Additional file 1: Fig. S9A). Of note, the prognostic value of the 3 gene-combination was superior to those obtained when combining two out of three genes (Additional file 1: Fig. S9B–D). A higher *PRNP*-*CTNNB1*-*NR3C1* score was also associated with a worse prognosis in the entire PETACC8 cohort (HR = 1.23, CI = 1.01–1.50, *p* = 0.04) (Additional file 1: Fig. S8E) or within each arm (Additional file 1: Fig. S8F, G). Altogether, these results exemplify the *PRNP*-*CTNNB1*-*NR3C1* axis as a poor prognosis trait of CRC.

## Discussion

**Fig. 8:**
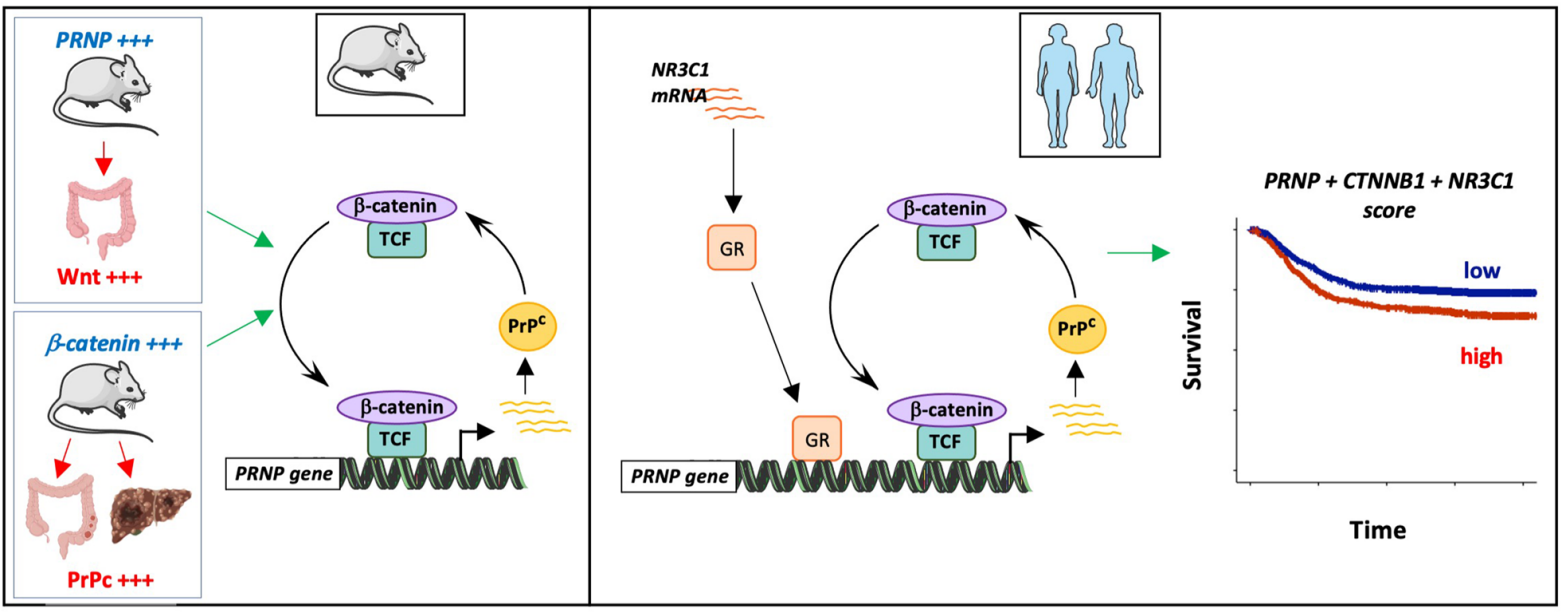
Graphical summary

This study uncovers a new gene regulatory network involving PrP^C^, the Wnt and Glucocorticoid signaling pathways that pertains to CRC progression and dismal outcome (Fig. 8) We have recently documented that PrP^C^ is overexpressed in the mesenchymal, poor-prognosis subtype of CRC, and have shed some mechanistic insight into how it contributes to the mesenchymal phenotype of CRC cells [9, 32]. Going one step further, we have provided the proof of concept that antibody-mediated neutralization of PrP^C^ may represent a novel therapeutic strategy to target mesenchymal CRC [10]. However, an integrated view of the mechanisms leading to the upregulation of PrP^C^ was lacking. Several cancer studies have documented an induction of PrP^C^ expression upon ER stress [45], hypoxia [46] or DNA damage [47]. From a molecular point of view, the *PRNP* promoter has been shown to be positively regulated by several transcription factors including sXBP1 [45], AP1 [48] as well as NFIL3 [49]. Here, we first uncovered that PrP^C^ is a downstream target of the canonical Wnt-β-catenin pathway through in silico and molecular analyses in several models of colon and liver cancer based on β-catenin overactivation. One major finding is that, in human, *PRNP* gene regulation is also highly modulated by glucocorticoid signaling. This observation fully fits in with the notion that the transcriptional response to Wnt-β-catenin signaling is highly context- and co-factor-dependent [30], and with the recent report that β-catenin partners with the GR to promote stemness properties of prostate cancer cells [50]. That PrP^C^ levels are sensitive to corticoids has been previously documented in neutrophils by Mariante et al. [51], but, to our knowledge, this is the first report that this also holds true in the context of cancer. Our results also align well with the current view that the specificity of gene expression regulation is highly dependent upon TF combinations [52]. In this respect, we may envision that additional regulatory factors beyond β-catenin and the GR combine with those to regulate *PRNP* gene expression. Potential candidates include SMADs, since they both interact with β-catenin [8] and the GR [53] and relay the action of TGFβ, itself known to induce the expression of PrP^C^ [9, 51]. Further exploration is required to obtain an integrated view of the complex regulation of the *PRNP* gene in cancer.

While the study from Mariante and colleagues was performed in mice, our results obtained in human cohorts and mouse models, combined with comparative genomics of the regulatory sequences in the *PRNP* promoter, suggest that some aspects of *PRNP* regulation are specific to human, as reported for its regulation by the FOXP2 transcription factor [54]. This finding is reminiscent of the divergence of the p53 gene regulatory network between mouse and human emphasized by Fischer [55]. Incidentally, the identification of apparent inconsistencies after interrogating mouse and human datasets has sparked our search for potential β-catenin co-factors and led us to focus on the GR, whose response element differs between the mouse *Prnp* and human *PRNP* promoters. Besides, regardless of the differences between mouse and human, our generation of a *PRNP*-overexpressing Apc mouse model has proven instrumental to uncover the occurrence of a vicious circle deriving from the positive feedback loop linking PrP^C^ to the Wnt signaling pathway. Thus, despite limitations inherent to mouse studies, molecular and histological examination of our autochthonous mouse model has successfully informed us on the deleterious impact of the cooperation between PrP^C^ and aberrant Wnt signaling. Such observations were made possible by the unique characteristics of our model of PrP^C^ overexpression, insuring proper regulation of expression and gain of function. Of note, *Tgfb1* features among the genes that are induced downstream from PrP^C^ (see Fig. 4F, K), suggesting that TGFβ1 may also fuel the vicious circle fostering *PRNP* gene expression, since we [9] and others [51] have documented a positive regulation of *PRNP* by TGFβ1.

Adding yet another layer of complexity, it is now recognized that the action of the GR can differ according to whether it is liganded or not [56]. This prompted us to include the GR target gene *TSC22D3* in our analyses, as a readout of GR activation. Given the reciprocal relations between stress and cancer [57, 58], an endogenous activation of the GR in CRC patients is very likely. Incidentally, we obtained prominent correlations between the expression of *TSC22D3* and that of *PRNP* (see Additional file 1: Fig. S6M) or the *PRNP*-*CTNNB1*-*NR3C1* score (*R* = 0.55, Spearman) in the IDEA France cohort, indicating that tumors with high PrP^C^ expression exhibit intrinsic GR activation. Furthermore, beyond the circulation, one source of glucocorticoids may be the tumor itself, as emphasized recently [59]. A major finding in the later study is that tumor-derived glucocorticoids exert a paracrine action on Tregs and foster tumor growth through Treg activation [59]. It can thus be surmised that the mobilization of the *PRNP-CTNNB1-NR3C1* axis in tumor cells is associated with the emergence of an immune-suppressive tumor microenvironment. Another potential deleterious trait connected to the *PRNP-CTNNB1-NR3C1* axis would be the interference with therapy-induced antitumor immunity, as the induction of *TSC22D3* in dendritic cells (DCs) by glucocorticoids was shown to block interferon responses and subsequent T cell activation [60]. Altogether, these different processes may account for the dismal prognosis exemplified in patients with a high *PRNP-CTNNB1-NR3C1* score.

Importantly, we obtained a set of observations that consistently conspire to define high *PRNP-CTNNB1-NR3C1* as a pejorative trait in CRC, in addition to its prognostic value. Indeed, we found the *PRNP-CTNNB1-NR3C1* score to be higher in CRC tumors of the CMS4 subtype, those with a pejorative CMS combination, or those associated with a higher risk of relapse based on TNM staging. One key finding is the very robust correlation between the *PRNP-CTNNB1-NR3C1* score and the mesenchymal score defined by [43], arguing that the *PRNP-CTNNB1-NR3C1* axis may be a hallmark of mesenchymal tumors.

## Conclusion

In summary, the integration of in silico, cellular, mouse and human data allowed us to uncover an unprecedented cooperation between Wnt, GR and PrP^C^ signaling, which perpetuates a vicious circle in CRC. We found that the regulation of PrP^C^ expression is not strictly transposable from mouse to human, but that the manipulation of PrP^C^ expression in mouse can nonetheless help uncover regulatory networks, which subsequently proved to have clinical value. Our work thus advocates for the implementation of multimodal approaches to decipher the complexity of CRC. Finally, given the premises of PrP^C^ targeting [10], this study may pave the way towards new therapeutic strategies to treat mesenchymal CRC.

### Supplementary Information

**Additional file 1.** Materials and methods.

## Data Availability

Materials, data, and protocols described in the manuscript will be made available upon reasonable request.

## Abbreviations

APP: Amyloid precursor protein
CMS: Consensus molecular subtype
CRC: Colorectal cancer
DEN: Dimethylnitrosamine
Dex: Dexamethasone
GR: Glucocorticoid receptor
GRE: GR responsive element
HR: Hazard ratio
ILK: Integrin linked kinase
OPC: Oligodendrocyte progenitor cell
PrP^C^: Cellular prion protein
TF: Transcription factor
WRE: Wnt-responsive element
