# Supplementary material for: Wnt, glucocorticoid and cellular prion protein cooperate to drive a mesenchymal phenotype with poor prognosis in colon cancer
Source: J Transl Med. 2024 Apr 8;22:337. doi: 10.1186/s12967-024-05164-0 (PMC11003154; doi:10.1186/s12967-024-05164-0)
Supplement: Supplementary file 1 — Additional file 1. Materials and methods. [file 12967_2024_5164_MOESM1_ESM.zip › Supplementary materials Apc-PRNP JTM_revised.docx]

**Supplementary materials to:**

**Wnt, glucocorticoid and cellular prion protein cooperate to drive a mesenchymal phenotype with poor prognosis in colon cancer**

by Sophie Mouillet-Richard et al.

Supplementary Materials and Methods

Legends to Supplementary Figures 1 to 9

Supplementary Tables 1 to 4

Supplementary references

**Supplementary** **Materials and methods**

***Cell culture and treatment***

All tissue culture reagents were from Invitrogen (Carlsbad, CA, USA). The human colon MDST8 and LoVo cell lines were purchased from Sigma and the SW480 cell line from the ATCC, which provided cell authentication. MDST8 cells were grown in DMEM with 10% fetal bovine serum (FBS). LoVo cells were grown in F-12K medium supplemented with 10% FBS. SW480 cells were grown in Leibovitz’s L-15 medium supplemented with 10% FBS. All cell lines were grown at 37 °C and 5% CO_2_ in a humidified incubator and regularly tested for mycoplasma contamination. For transient siRNA-mediated silencing, cells were transfected with siRNA sequences (30 nM) (Thermo-Scientific, Waltham, MA, USA or sequences in [1]) as in [1]. For PrP^C^ overexpression, LoVo cells were transfected with 2.5 µg of the pcDNA3-Prnp plasmid that expresses mouse *Prnp* (kind gift of Pr. Sylvain Lehmann) using the Lipofectamine 3000 reagent according to the manufacturer’s instructions (Invitrogen) as in [1]. The corresponding empty vector was used as control. Treatment with dexamethasone (D2915) (Sigma-Aldrich, St. Louis, MO, USA) was carried out at a final concentration of 1 µM for 24h.

***Animal experiments***

All in vivo experiments were performed according to French legal regulations and were approved by the Committee on the Ethics of Animal Experiments from INRAE (Permit number HCB n°6460 and 6461, agreement 2877 and APAFiS #27387 and 16420). Mice were housed in individually ventilated cages with enrichment materials, under a 12-hour light-dark cycle and in a controlled temperature environment (21°C) following the guidelines and recommendations of the Federation of European Laboratory Animal Science Associations. All mice were fed ad libitum with a standard laboratory chow diet (65% carbohydrate, 11% lipids, and 24% proteins). Mouse models of conditional Apc inactivation include *VilCreER*^T2^*Apc*^fl/+^ [2] and *TTRCreER*^T2^*Apc*^fl/fl^ [3,4] mice. For PrP^C^ overexpression, we exploited Tg650 mice that display a 5 copy-insertion of an artificial chromosome containing the human *PRNP* gene and its regulatory elements [5]. They do not spontaneously develop tumours and have a normal lifespan. We may also note that PrP^C^ is a highly conserved protein and that PrP^C^ from different species can functionally substitute for each other [1]. Mice (both male and female) have been exposed to tamoxifen from the age of 2 months. In the colon cancer model, mice were treated by 4 injections of tamoxifen (T5648, Sigma-Aldrich), 1 per day for 4 days (1 mg, ICN) in corn oil IP, followed by cycles of one injection per month until sign of illness. Results from experiments carried out in *VilCreER*^T2^*Apc*^fl/+^ mice crossed with *PRNP*-overexpressing Tg650 mice are displayed in Figures 4 and 5 and Supplementary Figures 3 and 4. In the liver cancer model, 2-month-old Apc^fl/fl^ male mice received a unique retro-orbital injection of 10^9^Vg Ad5-Cre adenovirus resulting in the deletion of Apc in 10 to 50% of the Apc^fl/fl^ hepatocytes and tumors within 4 to 6 months, as previously described [6]. Livers tumours with N-Nitrosodiethylamine (DEN) was obtained by intraperitoneal injection of 0.25mg DEN in 14-day-old male mice (Tolba, Laboratory Animals 2015). For hepatocyte sorting, retro-orbital injections of 1.5x10^9^ particles of an Ad5-Cre-GFP adenovirus were performed on two-month-old Apc^fl/fl^ mice and GFP-positive hepatocytes were sorted with an ARIA3 (BD). GFP-negative cells or GFP+ cells from Apc^wt/wt^ mice were used as control cells with no activation of β-catenin. Results from experiments carried out in liver cancer models are displayed in Figure 6 and Supplementary Figure 5. These results correspond to either new qRT-PCR experiments, new ATACseq data or re-analysis of previously generated ChIPseq data [3], RNAseq data [4,7].

***Isolation of total RNA and RT-PCR analysis***

Total RNA was isolated by using the RNeasy (cell lines) or RNeasy mini (mouse tissue) extraction kit (Qiagen, Limburg, Netherlands), as per manufacturer's instructions. For reverse transcriptase-polymerase chain reaction (RT-PCR) analysis, first-strand cDNA was synthesized with oligo(dT) primer and random hexamers, using the PrimeScript reverse Transcriptase kit (TaKaRa, Shiga, Japan) according to the manufacturer's protocol. Real-time PCR was performed using Absolute QPCR SYBR Green ROX Mix (Thermo-Scientific, Waltham, MA, USA) on a QuantStudio (Life Technologies Corporation, Carlsbad, CA, Etats-Unis). Real-time PCR analyses were performed with the SDS software 2.3 (Applied Biosystems). Primers used for the PCR reactions are shown on Supplementary Table S4. Results are expressed as a relative quantification of a target gene transcript normalized to the *RPL13A* (human cell lines) or 18s (mouse tissue) housekeeping gene using the ∆∆Ct method.

***Histology and immunohistochemistry.***

Immediately after the mouse was killed, its entire gastrointestinal tract was removed, splayed open along its length and rolled up from the proximal to distal end to form a ‘Swiss roll’. Tissues were then fixed by incubation in 4% formol overnight at 4 °C and were embedded in paraffin wax. Haematoxylin/eosin staining was carried out on 3-μm paraffin sections. For immunohistochemistry, 5-μm sections were treated with 3% hydrogen peroxide for 15 min at room temperature. Antigen was retrieved by boiling in Tris/EDTA (pH 8) in a microwave pressure cooker (EZ retriever, Biogenex). Sections were incubated in blocking solution for 20 min at room temperature. The sections were then incubated overnight at 4 °C with primary antibodies diluted in blocking solution. The primary antibody used was directed against β-catenin (Transduction Laboratories, 610154, 1:50). Specific binding was detected with a biotinylated secondary antibody and ABC reagent (Vector), or with Envision-HRP (Dako). The signal was developed with DAB (Vector).

Histological slides were scanned with a x40 objective and visualized using Caseviewer viewing software. All images were reviewed and annotated by an expert pathologist.

***ChIPseq, ATACseq and promoter analyses***

Data from the GSE156083 [8] and GSE35213 [3] datasets as well as those from the ENCODE database were analysed with the Integrative Genome Viewer (IGV) [9]. For promoter analysis, the sequences of the human *PRNP* and mouse *Prnp* gene promoters were retrieved from the Eucaryotic Promoter Database (EPD) [10] and analysed for TCF7L2 and NR3C1 binding motifs using the JASPAR database [11]. Comparison between the human and mouse sequences were carried out using the Comparative Genomics tools from Ensembl [12]. For ATAC-seq experiments, 50,000 isolated hepatocytes were transposed for 30min in 50μL reaction mix containing 4.5μL transposase (Illumina kit #FC-121-103) and 0.1% digitonin (adapted from [13]). After transposition, the following steps were performed according to the initial protocol published by Buenrostro et al. [14]. Libraries were controlled using a 2100 Bioanalyzer, and an aliquot of each library was sequenced at low depth onto a MiSeq platform to control duplicate level and estimate DNA concentration. Each library was then paired-end sequenced (2 × 100 bp) on a HiSeq instrument to get 40 million read pairs on average. As ATACseq libraries are composed in large part of short genomic DNA fragments, and in order to reduce costs, we next decided to sequence our recent libraries on a Nextseq instrument (2 x 38bp). Our analysis showed that reducing read length to 38bp does not affect mapping efficiency. Reads were first cleaned using trimmomatic (removing of adaptors and low-quality bases). Trimmed reads are then aligned to the mouse genome (mm9) using Bowtie2 with the parameter -X2000, and with 2-mismatches permitted in the seed (default value). The -X2000 option allows the fragments < 2kb to align. Duplicated reads were removed with picard-tools. Resulted bam datasets were then converted to BigWig, a coverage track adapted to visualize datasets in UCSC Genome Browser or IGV. Conversion was performed using bamCoverage command from deepTools with the parameters --binSize 10 --normalizeUsing RPKM --extendReads. The parameter --normalizeUsing RPKM is used to normalize each dataset. We selected the normalization method based on RPKM (Reads Per Kilobase per Million mapped reads), which calculates the number of reads per bin / number of mapped reads (in millions). The parameter --extendReads allows the extension of reads to fragment size. The default value is estimated from the data (mean of the fragment size of all mate reads). ATACseq data has been deposited with accession number GSE242267.

***3’RNAseq and preparation of data for patient cohorts***

Tumor RNA was extracted from macrodissected FFPE punch biopsy using the RNeasy ™ FFPE kit (Qiagen) in IDEA France trial and from macrodissected formalin-fixed, paraffin-embedded (FFPE) tissue sections using the QIAsymphony ™ RNA kit (Qiagen) in PETACC8 trial. The PolyA-RNAseq library preparation protocols were performed using 400ng of template RNA and the QuantSeq 3’mRNA-Seq Kit FWD for Illumina (Lexogen™) according to the manufacturer's instructions. Libraries were sequenced on NovaSeq6000 (Illumina). FASTQ RNA-seq files were mapped using STAR aligner 2.7.9a [15] and raw read counts were obtained using Rsubread R package [16]. Prior to mapping, the genome index was built on GRCh38.p13 human genome. Only samples with more than 10000 genes detected were kept for further analyses. Batch correction was performed using Combat_seq function from the sva R package [17]. Mitochondrial and housekeeping genes were removed from the raw count matrix. Then, for each sample, we scaled the corresponding vector of raw counts: counts were divided by the total number of counts of the vector and multiplied by the median of total number of counts across samples. Finally, the scaled data were log2 transformed.

**Supplementary figures legends**

**Supplementary Figure 1.** Alignment of the promoter sequences of the *PRNP* gene reveals species differences. **(a)** Promoter sequences of the human, macaque, mouse and rat *PRNP* / *Prnp* gene were compared using the Ensembl comparative genomics tool. Sequences highlighted in red, green and blue correspond to the conserved WRE, non-conserved rodent GRE and non-conserved primate GRE, respectively. **(b)** Predicted NR3C1 binding site within the mouse *Prnp* gene promoter.

**Supplementary Figure 2.** Impact of the modulation of *PRNP* expression on the expression of *AXIN2* in CRC cell lines. **(a-b)** Relative mRNA levels of *PRNP* and *AXIN2* in *PRNP*-silenced versus control MDST8 **(a)** or SW480 **(b)** cells, as determined in qPCR analysis. **(c)** Relative mRNA levels of *AXIN2* in PrP^C^-overexpressing versus control LoVo cells, as determined in qPCR analysis. Results are expressed as means of n=2 independent triplicates of cell preparations ± s.e.m. (* *p*< 0.05, ** *p*< 0.01, *** *p*< 0.001, Student’s t-test, except for **(c)** Wilcoxon rank-sum test).

**Supplementary Figure 3.** Generation of a mouse model combining *Apc* inactivation and human *PRNP* overexpression. **(a)** Schematic diagram representing the crossing of *VilCreER*^T2^*Apc*^fl/+^ and *PRNP*^+/+^ mice. **(b-j)** qRT-PCR analysis of the expression of *PRNP* **(b)**, *App* **(c)**, *Bace1* **(d)**, *Dkk3* **(e)**, *Pdgfc* **(f)**, *Tgfb1* **(g)**, *Axin2* **(h)**, *Lgr5* **(i)** and *Ccnd1* **(j)** in normal tissue from control mice (n=2 Cre-negative, n= 8 Tamoxifen-untreated) and *VilCreER*^T2^*Apc*^fl/+^*PRNP*^+/-^ mice (n= 8 samples) demonstrating the absence of effect of Tamoxifen in normal tissue. Mice experiments were conducted as described in Supplementary materials and methods section.

**Supplementary Figure 4.** Impact of PrP^C^ overexpression in normal tissue of *VilCreER*^T2^*Apc*^fl/+^ mice. **(a-i)** Boxplots showing the mRNA levels of human *PRNP* **(a)**, and mouse *App* **(b)**, *Bace1* **(c)**, *Dkk3* **(d)**, *Pdgfc* **(e)** *Tgfb1* **(f)**, *Axin2* **(g)**, *Lgr5* **(h)** and *Ccnd1* **(i)** in normal tissue from *VilCreER*^T2^*Apc*^fl/+^-*PRNP*^+/-^ (PRNP_het) (n = 8 samples) or *VilCreER*^T2^*Apc*^fl/+^-*PRNP*^+/+^ (PRNP_hom) mice (n = 7 samples) as measured through qRT-PCR. Mice experiments were conducted as described in Supplementary materials and methods section.

**Supplementary Figure 5.** Additional analyses in mouse liver cancer models. **(a-b)** Boxplots showing the mRNA levels of mouse *Prnp* **(a)** and *Axin2* **(b)** in liver tumours (n = 15) versus control liver tissues (n = 15) from mice exposed to DEN, as measured through qRT-PCR. **(c-d)** Analysis of our PRJEB44400 dataset reveals gradual increased *Axin2* **(c)** and *Ccnd1* **(d)** expression in differentiated and undifferentiated liver tumours from mutant *Ctnnb1* and *Apc* mice.

**Supplementary Figure 6.** *PRNP* and its targets are over-represented in poor-prognosis subtypes of CRC in the IDEA-France randomized clinical trial. **(a-h)** Relative *PRNP* **(a)**, *BACE1* **(b)**, *DKK3* **(c)**, *PDGFC* **(d)**, *NR3C1* **(e)**, *TSC22D3* **(f)**, *AXIN2* **(g)** and *LGR5* **(h)** expression according to the CMS classification in the IDEA France cohort. **(i-m)** Scatter plots the correlation between *PRNP* and *BACE1* **(i)**, *DKK3* **(j)**, *PDGFC* **(k)**, *NR3C1* **(l)**, *TSC22D3* **(m)** expression levels in the IDEA France cohort. **(n-u)** Relative *PRNP* **(n)**, *BACE1* **(o)**, *DKK3* **(p)**, *PDGFC* **(q)**, *NR3C1* **(r)**, *TSC22D3* **(s)**, *AXIN2* **(t)** and *LGR5* **(u)** expression according to poor-prognosis CMS combination in the IDEA France cohort.

**Supplementary Figure 7.** *PRNP* and its targets are over-represented in poor-prognosis subtypes of CRC in the PETACC8 randomized clinical trial. **(a-h)** Relative *PRNP* **(a)**, *BACE1* **(b)**, *DKK3* **(c)**, *PDGFC* **(d)**, *NR3C1* **(e)**, *TSC22D3* **(f)**, *AXIN2* **(g)** and *LGR5* **(h)** expression according to the CMS classification in the PETACC8 cohort. **(i-m)** Scatter plots the correlation between *PRNP* and *BACE1* **(i)**, *DKK3* **(j)**, *PDGFC* **(k)**, *NR3C1* **(l)**, *TSC22D3* **(m)** expression levels in the PETACC8 cohort. **(n-u)** Relative *PRNP* **(n)**, *BACE1* **(o)**, *DKK3* **(p)**, *PDGFC* **(q)**, *NR3C1* **(r)**, *TSC22D3* **(s)**, *AXIN2* **(t)** and *LGR5* **(u)** expression according to poor-prognosis CMS combination in the PETACC8 cohort.

**Supplementary Figure 8.** The *PRNP*-dependent axis is over-represented in poor-prognosis subtypes of CRC in the PETACC8 randomized clinical trial and predicts dismal outcome. **(a-c)** Relative *PRNP*-*CTNNB1*-*NR3C1* score according to the CMS classification **(a)**, CMS combination **(b)** or TNM risk **(c)** in the PETACC8 cohort. (**d**) Table summarizing the characteristics of patients belonging to the low or high *PRNP*-*CTNNB1*-*NR3C1* score group of patients in the PETACC8 cohort. **(e)** Kaplan-Meier curve comparing time to recurrence in patients with a low or high *PRNP*-*CTNNB1*-*NR3C1* score in the entire PETACC8 cohort. (**f**) Kaplan-Meier curve comparing time to recurrence in patients with a low or high *PRNP*-*CTNNB1*-*NR3C1* score in the subgroup of patients from the PETACC8 cohort having received FOLFOX. (**g**) Kaplan-Meier curve comparing time to recurrence in patients with a low or high *PRNP*-*CTNNB1*-*NR3C1* score in the subgroup of patients from the PETACC8 cohort having received FOLFOX plus cetuximab.

**Supplementary Figure 9.** The *PRNP*-*CTNNB1*-*NR3C1* score predicts TTR in FOLFOX-treated patients of the IDEA France cohort and has superior clinical value than *PRNP*-*CTNNB1*, *CTNNB1*-*NR3C1* and *PRNP*-*NR3C1* scores. Kaplan-Meier curve comparing time to recurrence in patients with a low or high *PRNP*-*CTNNB1*-*NR3C1* score in the subgroup of patients from the IDEA France cohort having received FOLFOX (**A**). Kaplan-Meier curves comparing time to recurrence in patients with low or high *PRNP*-*CTNNB1* (**B**), *CTNNB1*-*NR3C1* (**C**) and *PRNP*-*NR3C1* (**D**) scores in the IDEA France cohort.

**Supplementary Table S1.**

Demographics of the IDEA France cohort.

**Table S1. IDEA France**

| **Variable** | **N = 1,248**^1^ |
| --- | --- |
| **Age** | 65 (58, 71) |
| **Gender** |  |
| Female | 532 (43%) |
| Male | 708 (57%) |
| **Risk** |  |
| pT1-pT3 and pN1 | 742 (60%) |
| pT4 or pN2 | 498 (40%) |
| **WHO performance status** |  |
| WHO 0 | 924 (75%) |
| WHO 1-2 | 316 (25%) |
| **CMS** |  |
| CMS1 | 179 (14%) |
| CMS2 | 423 (34%) |
| CMS3 | 266 (21%) |
| CMS4 | 380 (30%) |
| **Duration of chemotherapy** |  |
| 3 Months | 611 (49%) |
| 6 Months | 629 (51%) |
| ^1^Median (IQR) or Frequency (%) | |

**Supplementary Table S2.**

Demographics of the PETACC8 cohort.

**Table S2. PETACC8**

| **Variable** | **N = 1,733**^1^ |
| --- | --- |
| **Age** | 60 (53, 67) |
| **Gender** |  |
| FEMALE | 746 (43%) |
| MALE | 987 (57%) |
| **Risk** |  |
| pT1-pT3 and pN1 | 891 (51%) |
| pT4 or pN2 | 841 (49%) |
| **Performance** |  |
| WHO 0 | 1,364 (82%) |
| WHO 1-2 | 307 (18%) |
| **CMS** |  |
| CMS1 | 309 (18%) |
| CMS2 | 635 (37%) |
| CMS3 | 362 (21%) |
| CMS4 | 412 (24%) |
| **Treatment arm** |  |
| FOLFOX | 858 (50%) |
| FOLFOX + cetuximab | 875 (50%) |
| ^1^Median (IQR) or Frequency (%) | |

**Supplementary Table S3.**

Baseline characteristics of patients belonging to the low or high *PRNP*-*CTNNB1*-*NR3C1* score group of patients in the IDEA France cohort.

**Table S3. IDEA France**

|  | | **PRNP_CTNNB1*_*NR3C1_score** | |  |
| --- | --- | --- | --- | --- |
| **Variable** | **Overall**, N = 1,248^1^ | **low**, N = 836^1^ | **high**, N = 412^1^ | **p-value**^2^ |
| **Age** | 65 (58, 71) | 65 (58, 71) | 64 (58, 70) | 0.12 |
| **Sex** |  |  |  | 0.27 |
| Female | 532 (43%) | 347 (42%) | 185 (45%) |  |
| Male | 708 (57%) | 483 (58%) | 225 (55%) |  |
| **Risk** |  |  |  | **0.003** |
| pT1-pT3 and pN1 | 742 (60%) | 521 (63%) | 221 (54%) |  |
| pT4 or pN2 | 498 (40%) | 309 (37%) | 189 (46%) |  |
| **WHO performance status** |  |  |  | 0.084 |
| WHO 0 | 924 (75%) | 606 (73%) | 318 (78%) |  |
| WHO 1-2 | 316 (25%) | 224 (27%) | 92 (22%) |  |
| **CMS** |  |  |  | **<0.001** |
| CMS1 | 179 (14%) | 124 (15%) | 55 (13%) |  |
| CMS2 | 423 (34%) | 369 (44%) | 54 (13%) |  |
| CMS3 | 266 (21%) | 223 (27%) | 43 (10%) |  |
| CMS4 | 380 (30%) | 120 (14%) | 260 (63%) |  |
| **CMS combination** |  |  |  | **<0.001** |
| bad | 233 (19%) | 105 (13%) | 128 (31%) |  |
| good | 1,015 (81%) | 731 (87%) | 284 (69%) |  |
| **Duration of chemotherapy** |  |  |  | 0.63 |
| 3 Months | 611 (49%) | 413 (50%) | 198 (48%) |  |
| 6 Months | 629 (51%) | 417 (50%) | 212 (52%) |  |
| ^1^Median (IQR) or Frequency (%) | | | | |
| 2Wilcoxon rank sum test; Pearson's Chi-squared test | | | | |

**Supplementary Table 4:** List of primers used

|  | Forward | Reverse |
| --- | --- | --- |
| hAXIN2 | TACACTCCTTATTGGGCGATCA | TTGGCTACTCGTAAAGTTTTGGT |
| hCTNNB1 | GATTTGATGGAGTTGGACATGGC | CCAGGTAAGACTGTTGCTGCCAG |
| hNR3C1 | TAGGTGCCAAGGATCTGGAG | AGAGTTTGGGAGGTGGTCCT |
| hPRNP | CGAGCTTCTCCTCTCCTCAC | GTTCCATCCTCCAGGCTTC |
| hRPL13A | CCTGGAGGAGAAGAGGAAAGAGA | GAGGACCTCTGTGTATTTGTCAA |
| hTSC22D3 | GGGAACTACCTGCATTTGGA | GTGCATCCGAGAAACAACCT |
| m18s | CGGCTACCACATCCAAGGAA | GCTGGAATTACCGCGGCT |
| mApp | GGTTCTGGGCTGACAAACAT | CAGTTTTTGATGGCGGACTT |
| mAxin2 | GGGGGAAAACACAGCTTACA | ACTGGGTCGCTTCTCTTGAA |
| mBace1 | ACCACCAACCTTCGCTTGCCC | AAGGGGTCGTGCCTGCTTGC |
| mCcnd1 | CAGAAGTGCGAAGAGGAGGTC | TACTCTTAGAGGCCACGAACAT |
| mDkk3 | TCAGGAGGAAGCTACGCTCAA | GTTCACCTCAGAGGACGTTTTAG |
| mLgr5 | CCTTGGCCCTGAACAAAATA | ATTTCTTTCCCAGGGAGTGG |
| mPdgfc | AGGTTGTCTCCTGGTCAAGC | CCTGCGTTTCCTCTACACAC |
| mPrnp | TTGGCAACGACTGGGAGGAC | GGACTCCTTCTGGTACTGGGTGA |
| mTgfb1 | TGGAGCCTGGACACACAGTA | GTTGGACAACTGCTCCACCT |

**Supplementary references**

1. Le Corre D, Ghazi A, Balogoun R, Pilati C, Aparicio T, Martin-Lannerée S, et al. The cellular prion protein controls the mesenchymal-like molecular subtype and predicts disease outcome in colorectal cancer. EBioMedicine. 2019;46:94–104.

2. Lévy J, Cacheux W, Bara MA, L’Hermitte A, Lepage P, Fraudeau M, et al. Intestinal inhibition of Atg7 prevents tumour initiation through a microbiome-influenced immune response and suppresses tumour growth. Nat Cell Biol. 2015;17:1062–73.

3. Gougelet A, Torre C, Veber P, Sartor C, Bachelot L, Denechaud P-D, et al. T-cell factor 4 and β-catenin chromatin occupancies pattern zonal liver metabolism in mice. Hepatology. 2014;59:2344–57.

4. Loesch R, Caruso S, Paradis V, Godard C, Gougelet A, Renault G, et al. Deleting the β-catenin degradation domain in mouse hepatocytes drives hepatocellular carcinoma or hepatoblastoma-like tumor growth. J Hepatol. 2022;77:424–35.

5. Béringue V, Le Dur A, Tixador P, Reine F, Lepourry L, Perret-Liaudet A, et al. Prominent and persistent extraneural infection in human PrP transgenic mice infected with variant CJD. PloS One. 2008;3:e1419.

6. Colnot S, Decaens T, Niwa-Kawakita M, Godard C, Hamard G, Kahn A, et al. Liver-targeted disruption of Apc in mice activates beta-catenin signaling and leads to hepatocellular carcinomas. Proc Natl Acad Sci U S A. 2004;101:17216–21.

7. Sanceau J, Poupel L, Joubel C, Lagoutte I, Caruso S, Pinto S, et al. DLK1/DIO3 locus upregulation by a β-catenin-dependent enhancer drives cell proliferation and liver tumorigenesis. Mol Ther. 2024;S1525-0016(24)00037-6.

8. Wan C, Mahara S, Sun C, Doan A, Chua HK, Xu D, et al. Genome-scale CRISPR-Cas9 screen of Wnt/β-catenin signaling identifies therapeutic targets for colorectal cancer. Sci Adv. 2021;7:eabf2567.

9. Robinson JT, Thorvaldsdóttir H, Winckler W, Guttman M, Lander ES, Getz G, et al. Integrative Genomics Viewer. Nat Biotechnol. 2011;29:24–6.

10. Meylan P, Dreos R, Ambrosini G, Groux R, Bucher P. EPD in 2020: enhanced data visualization and extension to ncRNA promoters. Nucleic Acids Res. 2020;48:D65–9.

11. Castro-Mondragon JA, Riudavets-Puig R, Rauluseviciute I, Lemma RB, Turchi L, Blanc-Mathieu R, et al. JASPAR 2022: the 9th release of the open-access database of transcription factor binding profiles. Nucleic Acids Res. 2022;50:D165–73.

12. Martin FJ, Amode MR, Aneja A, Austine-Orimoloye O, Azov AG, Barnes I, et al. Ensembl 2023. Nucleic Acids Res. 2023;51:D933–41.

13. Corces MR, Buenrostro JD, Wu B, Greenside PG, Chan SM, Koenig JL, et al. Lineage-specific and single-cell chromatin accessibility charts human hematopoiesis and leukemia evolution. Nat Genet. 2016;48:1193–203.

14. Buenrostro JD, Wu B, Chang HY, Greenleaf WJ. ATAC-seq: A Method for Assaying Chromatin Accessibility Genome-Wide. Curr Protoc Mol Biol. 2015;109:21.29.1-21.29.9.

15. Dobin A, Davis CA, Schlesinger F, Drenkow J, Zaleski C, Jha S, et al. STAR: ultrafast universal RNA-seq aligner. Bioinforma Oxf Engl. 2013;29:15–21.

16. Liao Y, Smyth GK, Shi W. The R package Rsubread is easier, faster, cheaper and better for alignment and quantification of RNA sequencing reads. Nucleic Acids Res. 2019;47:e47.

17. Zhang Y, Parmigiani G, Johnson WE. ComBat-seq: batch effect adjustment for RNA-seq count data. NAR Genomics Bioinforma. 2020;2:lqaa078.
