# Supplementary figures and images for: Wnt, glucocorticoid and cellular prion protein cooperate to drive a mesenchymal phenotype with poor prognosis in colon cancer
Source: J Transl Med. 2024 Apr 8;22:337. doi: 10.1186/s12967-024-05164-0 (PMC11003154; doi:10.1186/s12967-024-05164-0)

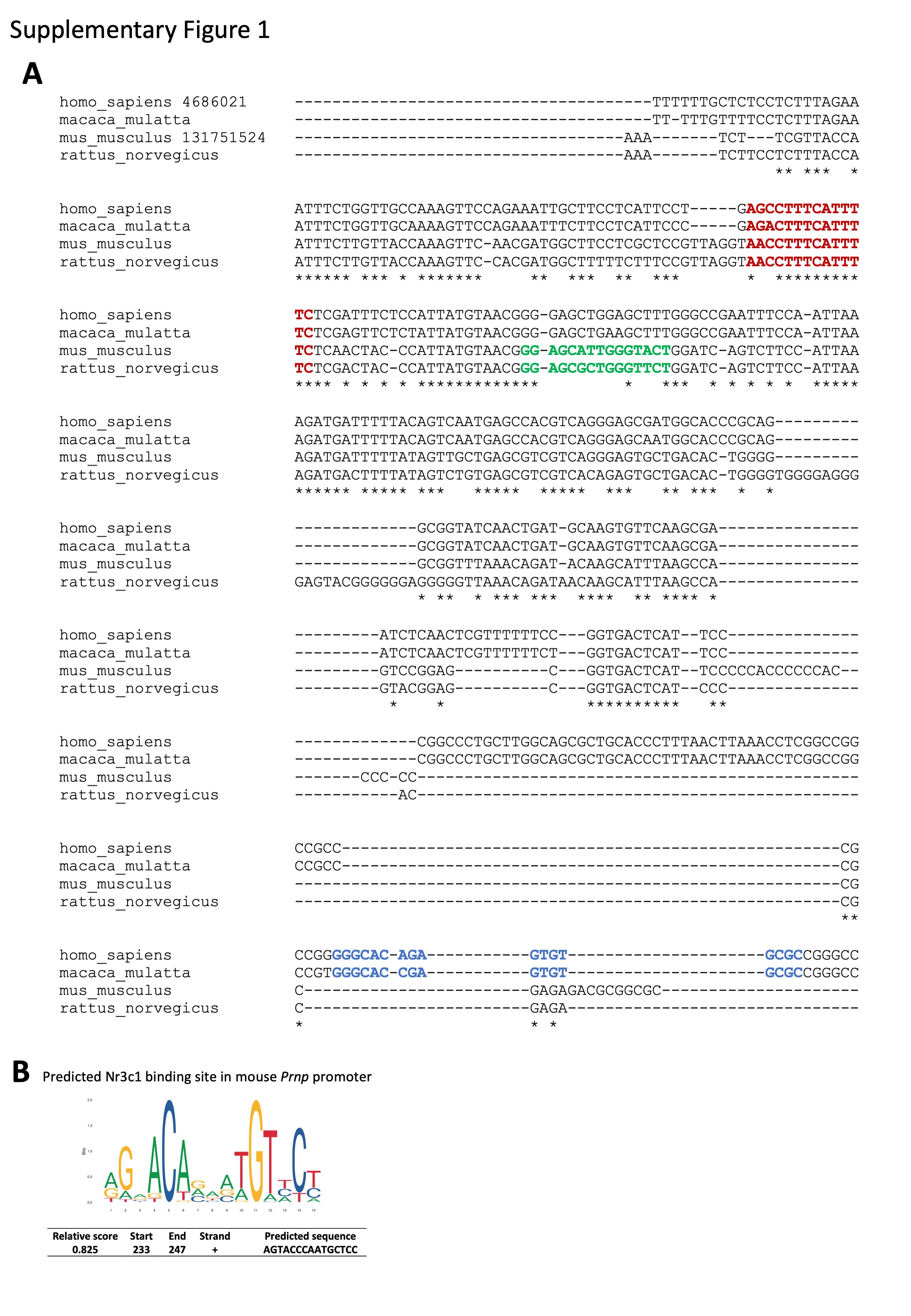

Supplement: Supplementary file 1 — Additional file 1. Materials and methods. [file 12967_2024_5164_MOESM1_ESM.zip › FigS1.jpeg]

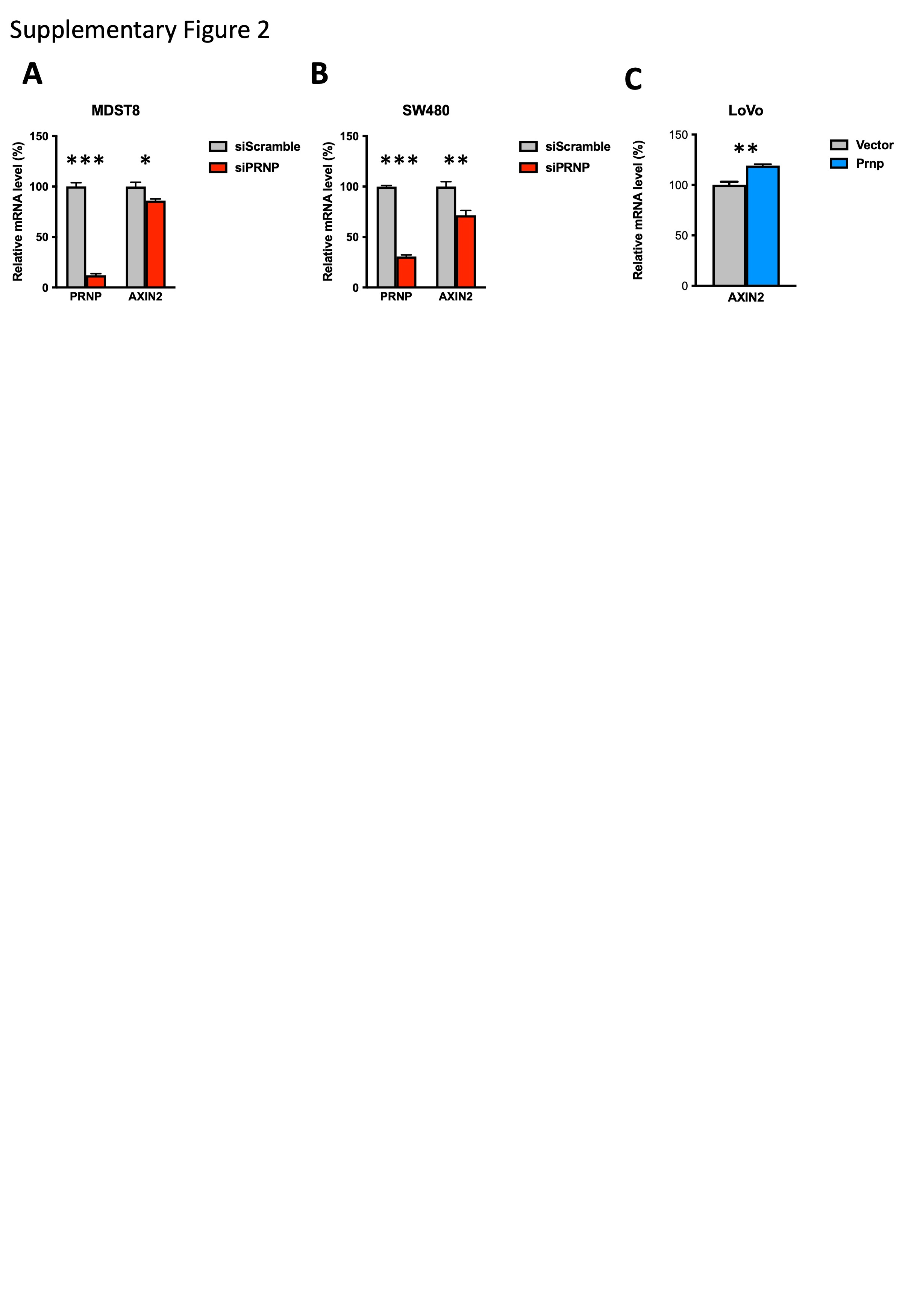

Supplement: Supplementary file 1 — Additional file 1. Materials and methods. [file 12967_2024_5164_MOESM1_ESM.zip › FigS2.jpeg]

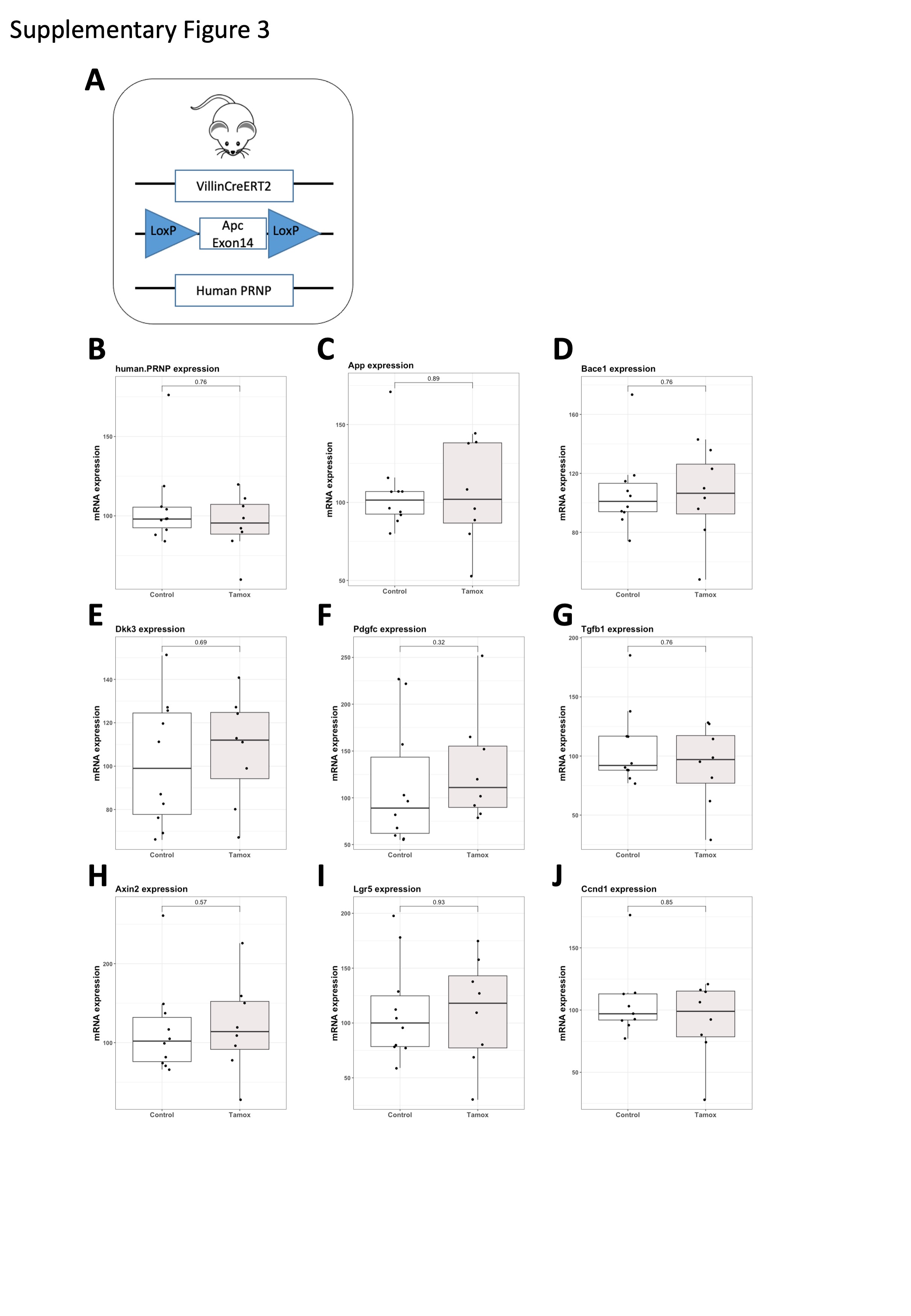

Supplement: Supplementary file 1 — Additional file 1. Materials and methods. [file 12967_2024_5164_MOESM1_ESM.zip › FigS3.jpeg]

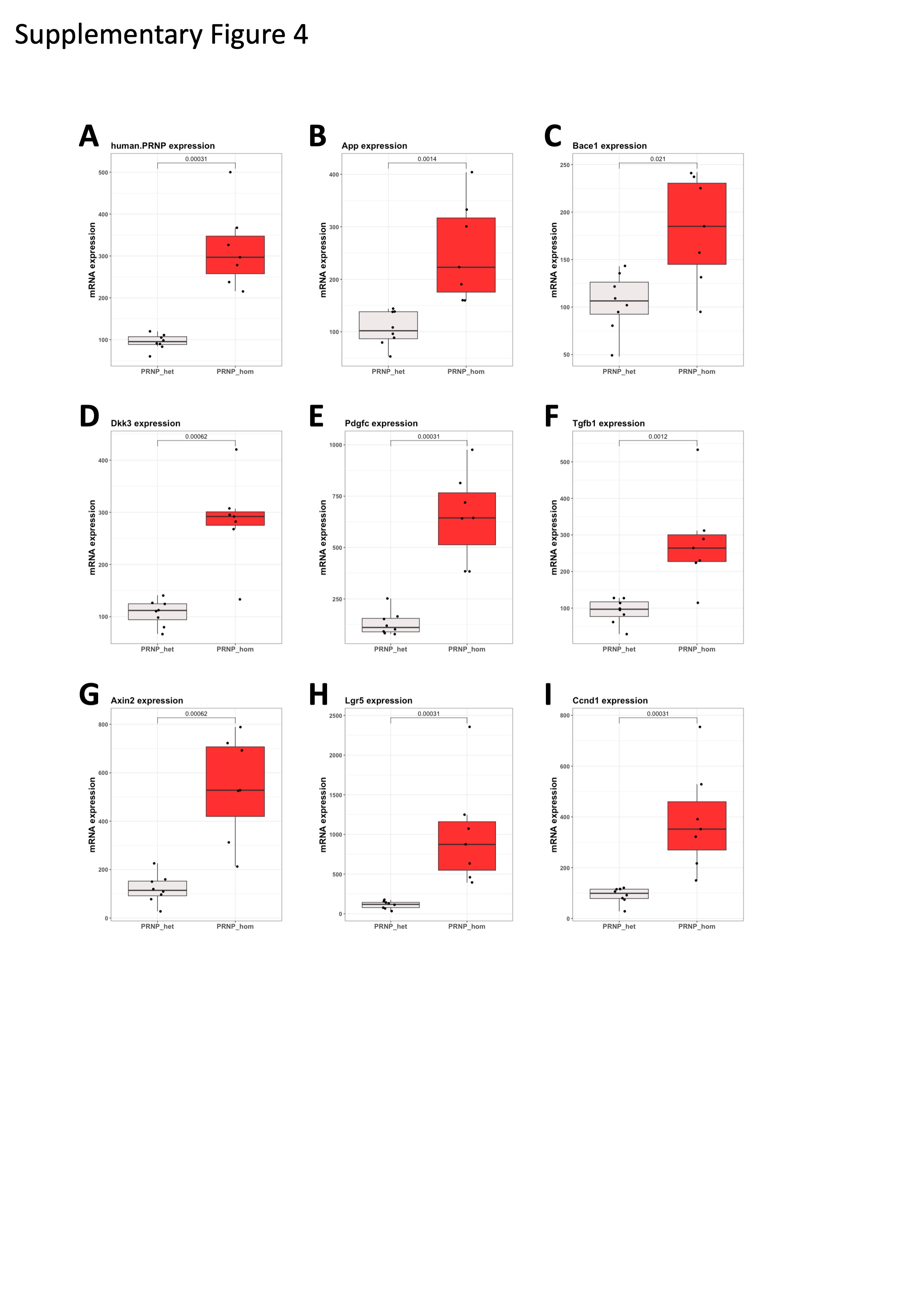

Supplement: Supplementary file 1 — Additional file 1. Materials and methods. [file 12967_2024_5164_MOESM1_ESM.zip › FigS4.jpeg]

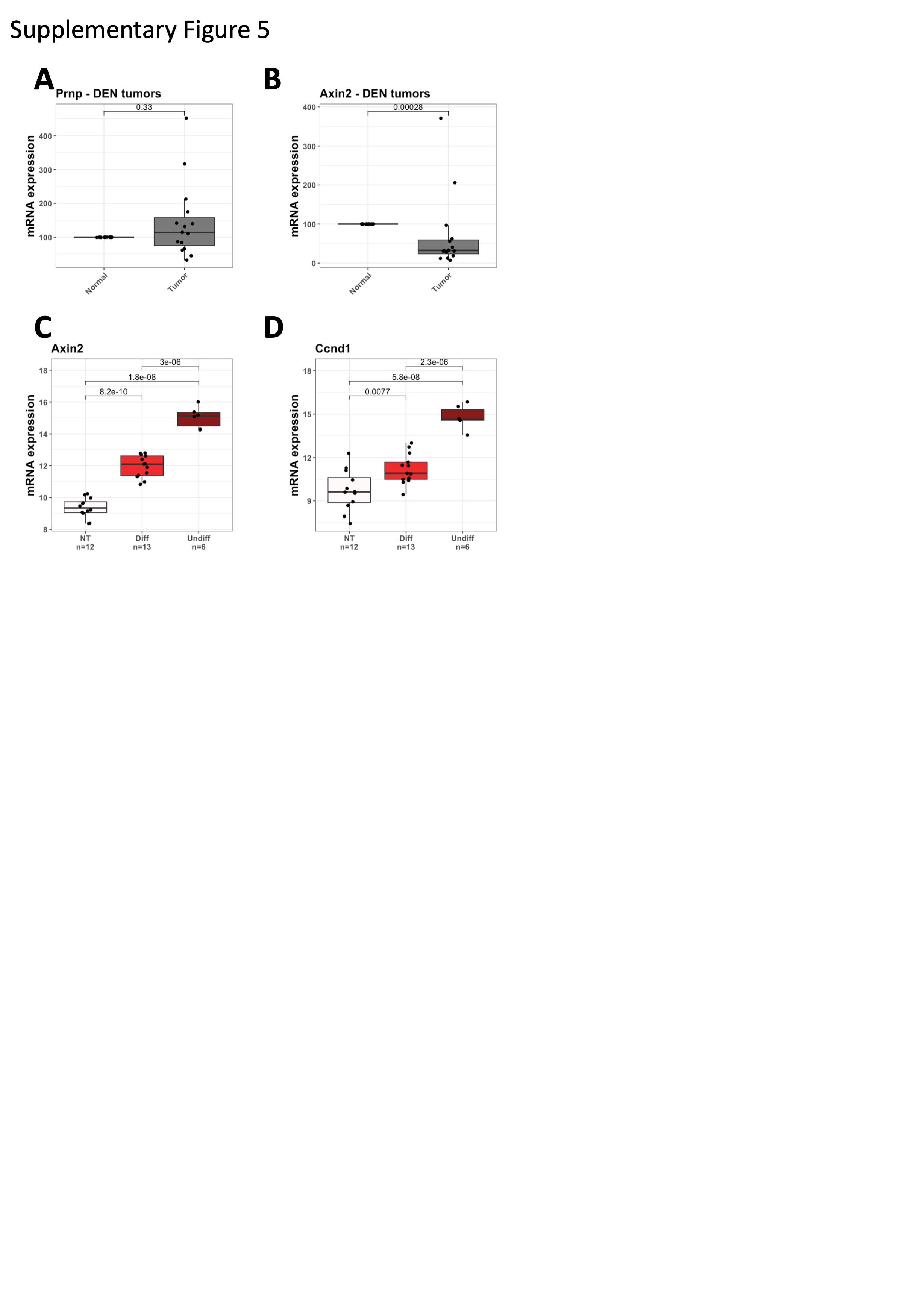

Supplement: Supplementary file 1 — Additional file 1. Materials and methods. [file 12967_2024_5164_MOESM1_ESM.zip › FigS5.jpeg]

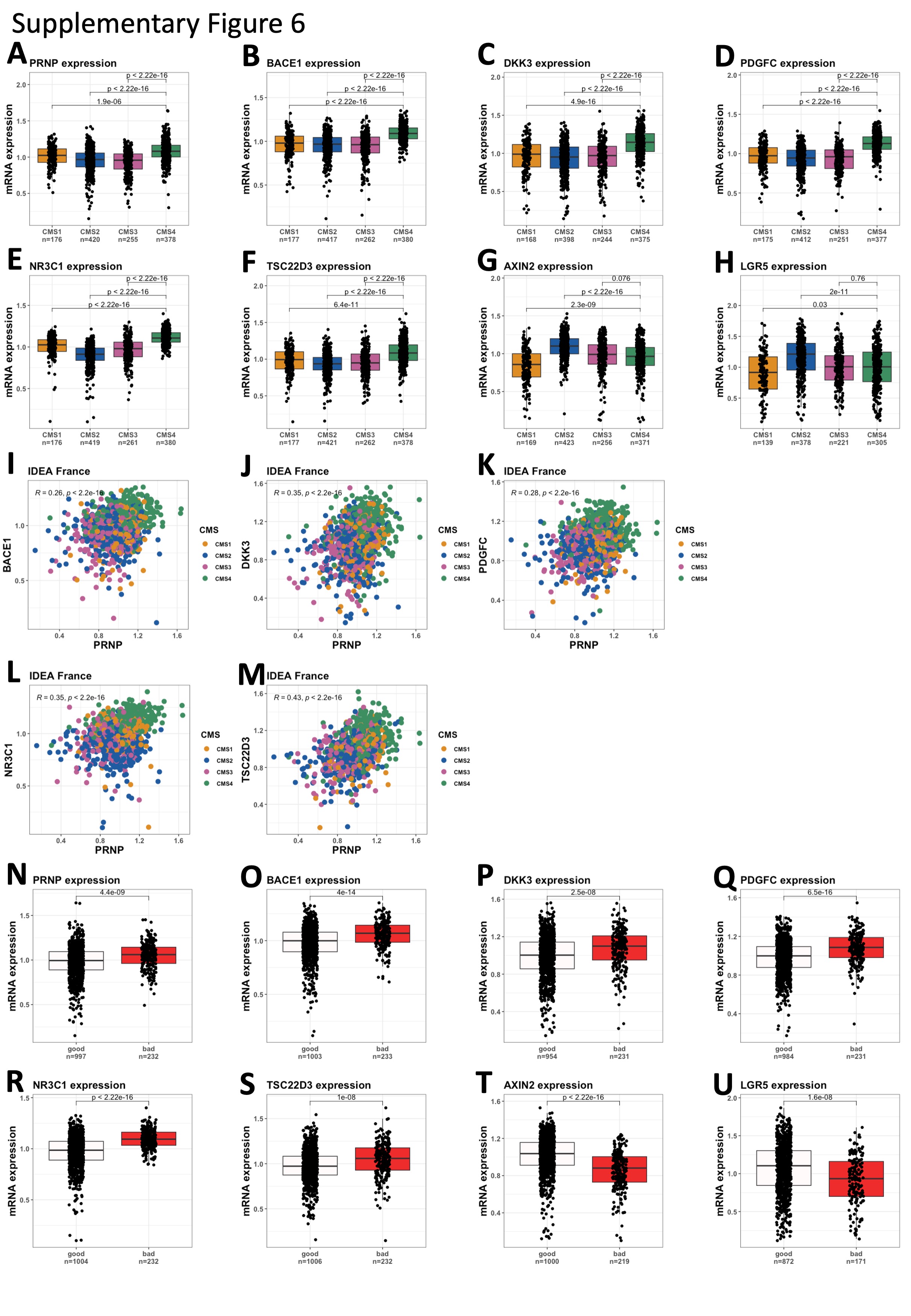

Supplement: Supplementary file 1 — Additional file 1. Materials and methods. [file 12967_2024_5164_MOESM1_ESM.zip › FigS6.jpeg]

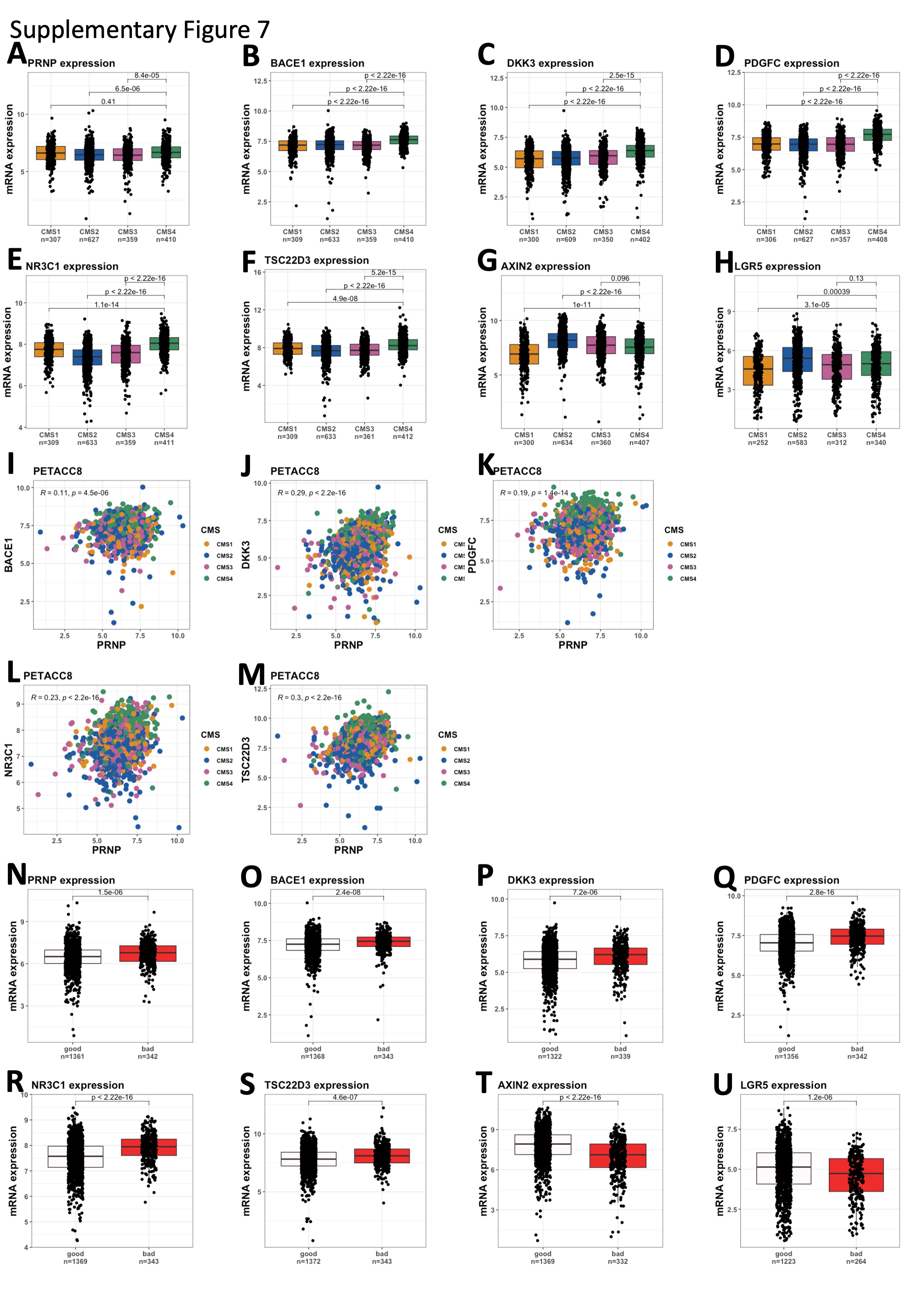

Supplement: Supplementary file 1 — Additional file 1. Materials and methods. [file 12967_2024_5164_MOESM1_ESM.zip › FigS7.jpeg]

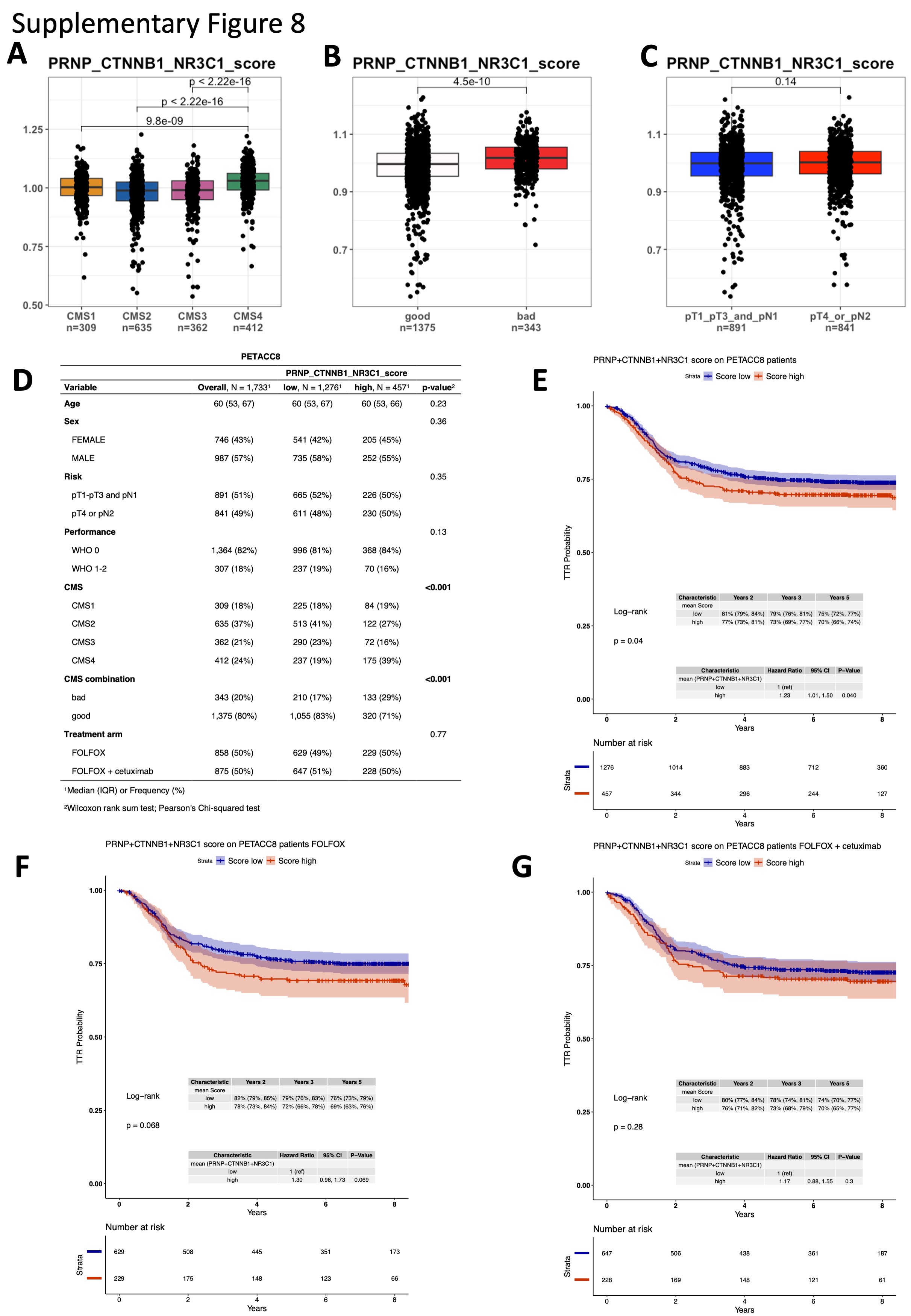

Supplement: Supplementary file 1 — Additional file 1. Materials and methods. [file 12967_2024_5164_MOESM1_ESM.zip › FigS8 rev.jpeg]

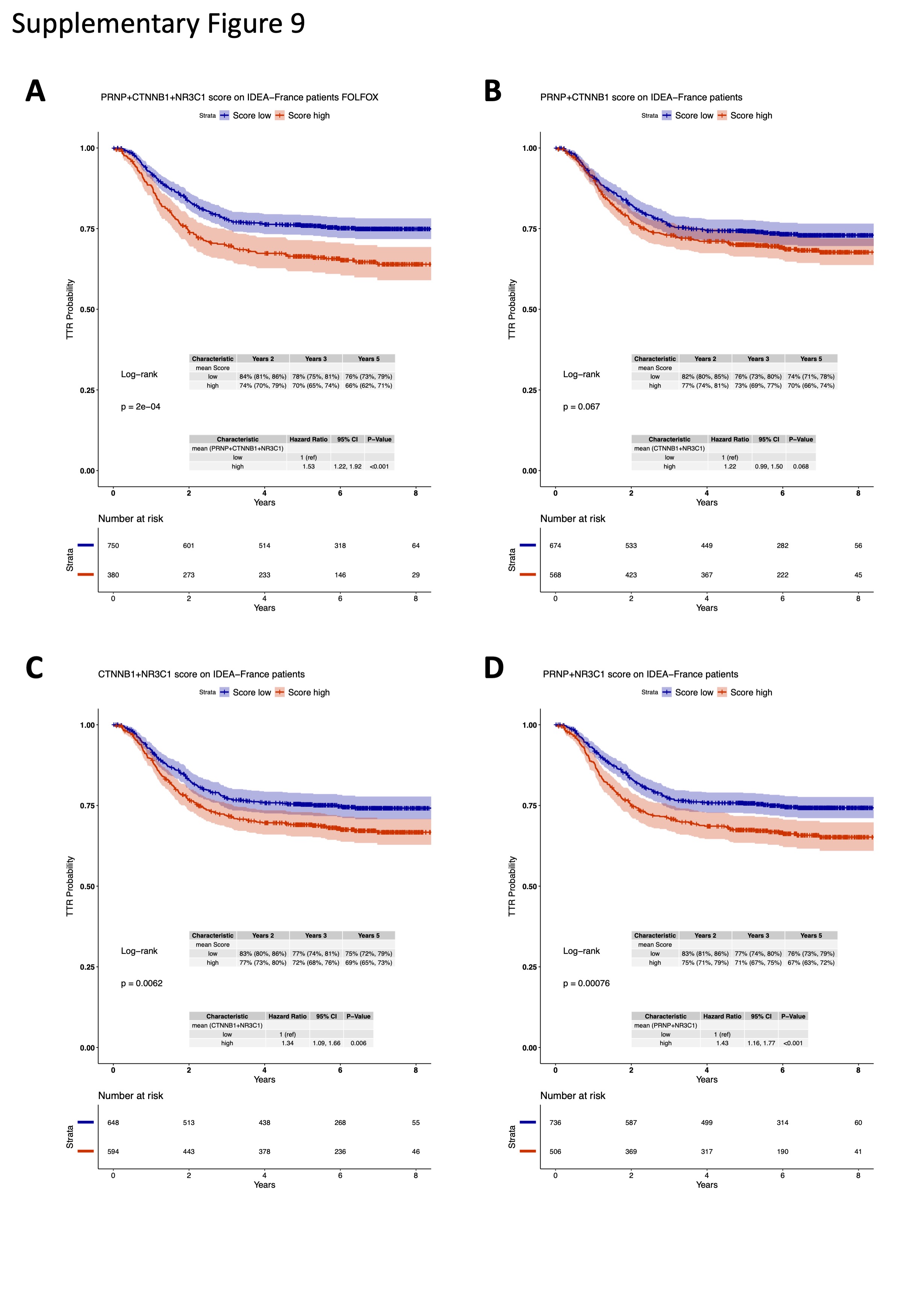

Supplement: Supplementary file 1 — Additional file 1. Materials and methods. [file 12967_2024_5164_MOESM1_ESM.zip › FigS9 rev.jpeg]
